# Control of successive unequal cell divisions by neural cell fate regulators determines embryonic neuroblast cell size

**DOI:** 10.1242/dev.200981

**Published:** 2024-02-05

**Authors:** Thomas W. Mullan, Terry Felton, Janis Tam, Osama Kasem, Tim J. Yeung, Nadin Memar, Ralf Schnabel, Richard J. Poole

**Affiliations:** ^1^ Department of Cell and Developmental Biology, University College London, London WC1E 6BT, UK; ^2^ Institut für Genetik, TU Braunschweig, D-38106 Braunschweig, Germany

**Keywords:** Asymmetric division, Neuronal specification, Proneural, *C*. *elegans*

## Abstract

Asymmetric cell divisions often generate daughter cells of unequal size in addition to different fates. In some contexts, daughter cell size asymmetry is thought to be a key input to specific binary cell fate decisions. An alternative possibility is that unequal division is a mechanism by which a variety of cells of different sizes are generated during embryonic development. We show here that two unequal cell divisions precede neuroblast formation in the C lineage of *Caenorhabditis elegans*. The equalisation of these divisions in a *pig-1/MELK* mutant background has little effect on neuroblast specification. Instead, we demonstrate that *let-19/MDT13* is a regulator of the proneural basic helix-loop-helix transcription factor *hlh-14/ASCL1* and find that both are required to concomitantly regulate the acquisition of neuroblast identity and neuroblast cell size. Thus, embryonic neuroblast cell size in this lineage is progressively regulated in parallel with identity by key neural cell fate regulators. We propose that key cell fate determinants have a previously unappreciated function in regulating unequal cleavage, and therefore cell size, of the progenitor cells whose daughter cell fates they then go on to specify.

## Teaser

**Highlighted Article:** 4D-lineage analysis and volumetric quantifications of the C lineage of *Caenorhabditis elegans* reveal that embryonic neuroblast size requires successive unequal cleavages that are regulated by key proneural transcription factors.

## INTRODUCTION

The development of multicellular organisms generates various cell types, each with its own unique molecular signature and specific cell size. Asymmetric cell division is one mechanism by which two daughter cells can acquire different fates (reviewed by Horvitz and Herskowitz, 1992). That asymmetric cell divisions can also generate daughters of unequal sizes has been known for over 100 years, since embryonic studies of leech (Whitman, 1878), *Ascaris* nematode (Boveri, 1899) and ascidians (Conklin, 1905) demonstrated asymmetric inheritance of developmental potentials and the establishment of cell lineages. However, why dividing cells regulate daughter cell size and whether this influences asymmetric fate specification remains unresolved.

The specification of asymmetric daughter cell fates during cell division is often established through asymmetric inheritance of fate determinants, such as proteins, RNA or cellular organelles (reviewed by Gönczy, 2008; Horvitz and Herskowitz, 1992; Knoblich, 2008; Sunchu and Cabernard, 2020). Studies focused on the *Caenorhabditis elegans* zygote and *Drosophila* neuroblasts have revealed that asymmetric segregation of key fate determinants depends on cortical polarity established by the conserved PAR network. Cortical polarity then influences asymmetric segregation of key cell fate-determining proteins, such as PIE-1, MEX-5 and MEX-6 in the *C. elegans* zygote and Prospero, Numb and Brat in *Drosophila* neuroblasts (reviewed by Gönczy, 2008; Loyer and Januschke, 2020; Neumüller and Knoblich, 2009; Rose and Gönczy, 2014; Sunchu and Cabernard, 2020). Importantly, the asymmetric segregation of Prospero into daughter cells controls fate but does not control cell size (Doe et al., 1991).

The molecular mechanisms regulating unequal cleavage fall into two categories (reviewed by Sunchu and Cabernard, 2020). One category is spindle dependent and employs asymmetric spindle positioning or pulling forces via a conserved G protein- and *lin-5/NuMa-*containing complex (Bowman et al., 2006; Nguyen-Ngoc et al., 2007; Siller et al., 2006; Srinivasan et al., 2003; Grill et al., 2001; Bonaccorsi et al., 2000; Fuse et al., 2003). The second is spindle independent and includes asymmetric cortical myosin flows and hydrostatic pressures generating apical expansion (Cabernard et al., 2010; Pham et al., 2019; Roubinet et al., 2017; Tsankova et al., 2017). In *C. elegans* both spindle-dependent and spindle-independent mechanisms are evident in the post-embryonic Q lineage. Anterior enrichment of cortical myosin is required for the unequal cleavage of the posterior daughter of the Q cell, Q.p, whereas the anterior daughter Q.a depends on spindle displacement (Ou et al., 2010). Both, however, depend on the kinase PIG-1, a PAR-1-like kinase (Cordes et al., 2006; Feng et al., 2013). PIG-1 also regulates a number of other unequal cell divisions in the *C. elegans* embryo (Liro et al., 2018; Pacquelet et al., 2015; Cordes et al., 2006; Wei et al., 2017), many of which produce a daughter that undergoes apoptosis and a sister cell that is either a neuroblast or neuron (Cordes et al., 2006; Wei et al., 2017). In the NSM neuroblast, PIG-1 acts upstream of myosin and may directly phosphorylate it (Wei et al., 2020). In *pig-1* mutants, loss of unequal division causes inappropriate survival of the cells normally fated to die, suggesting that cell size itself may affect the acquisition of apoptotic fate (Cordes et al., 2006). Inactivation of myosin in Q.a also leads to symmetric daughter cell size and daughter cell fate (Ou et al., 2010). In *Drosophila* neuroblasts and the one-cell *C. elegans* zygote, manipulation of spindle asymmetry or asymmetric pulling forces symmetrises daughter cell size as well as disrupting daughter cell fates (Cabernard and Doe, 2009; Fuse et al., 2003; Jankele et al., 2021). However, it is unclear to what extent these manipulations affect cortical polarity and/or asymmetric segregation.

Many organisms, including nematodes and flies, have an indirect programme of development in which no cell growth occurs in the embryo and the majority of cell and organismal growth is confined to the post-embryonic larval stages (reviewed by O'Farrell, 2004). Yet, at the end of embryogenesis, the larvae produced hatch with fully developed tissues containing cells of a variety of different sizes appropriate to their function. This raises an alternative hypothesis that the regulation of unequal cleavage may be used to generate cells of different sizes during development, particularly in situations where no cell growth is possible. Here, we show that DVC neuroblast formation in the *C. elegans* C lineage, the specification of which requires the expression of the proneural basic helix-loop-helix (bHLH) transcription factor-encoding gene *hlh-14* (Poole et al., 2011), is preceded by two distinctly unequal cleavages. We find that PIG-1 regulates these unequal cleavages, yet symmetrisation of daughter cell size in this mutant background has little effect on the expression of *hlh-14* in the DVC neuroblast, its specification, or the production of the DVC neuron. We reveal that the Mediator complex component LET-19 is a regulator of *hlh-14* and find that both LET-19 and HLH-14 concomitantly regulate the acquisition of neuroblast identity and neuroblast cell size, the latter via the regulation of unequal cleavage. Thus, cell size does not direct proneural gene expression. Instead, cell size is regulated in parallel with terminal identity to coordinate the cellular and molecular aspects of DVC specification. We propose that the regulation of unequal cleavage by key cell fate determinants is a mechanism to generate cell size differences in the absence of cell growth.

## RESULTS

### Two unequal cleavages precede *hlh-14 e*xpression in the DVC neuroblast

**Fig. 1.**
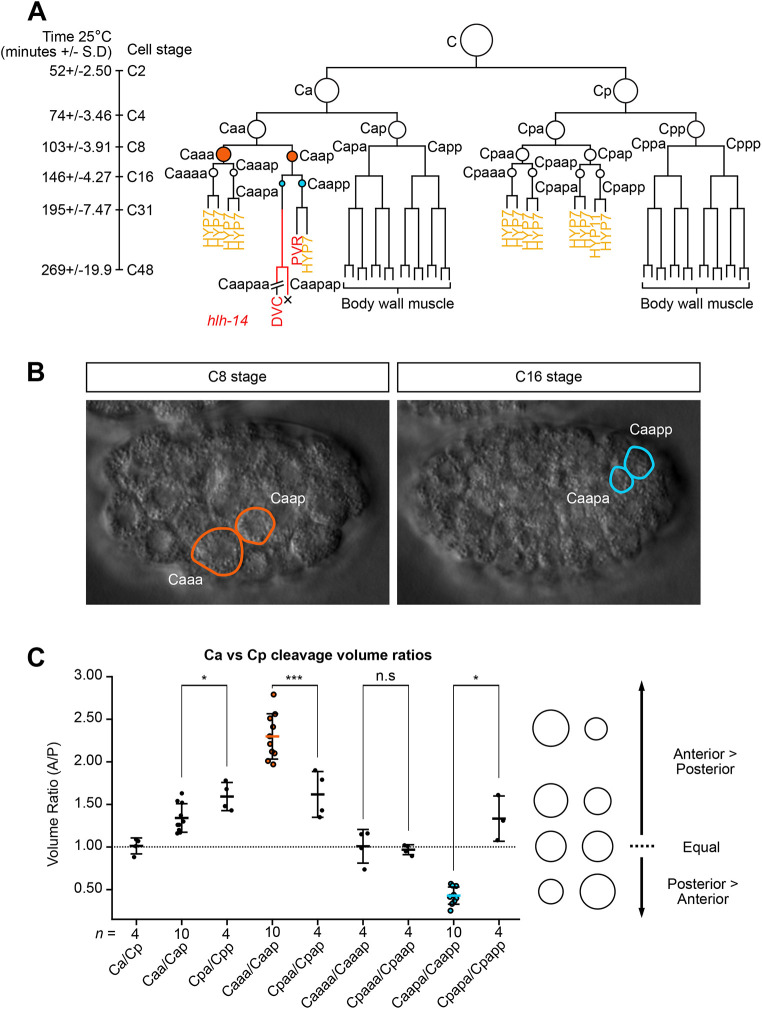
**Two dramatically unequal cleavages precede *hlh-14* expression in the C lineage.** (A) Diagram of the C lineage. Branch lengths indicate division timings, circles indicate relative cell size and X indicates cell death. Caa daughters are indicated in orange, Caap daughters in blue. Red lines indicate the timing of *hlh-14 [gmIs20]* expression. Red letters indicate neuronal fate; yellow letters indicate hypodermal fate. Fading of *hlh-14* prior to terminal fate differentiation is indicated. Division timing at 25°C is indicated in min±s.d.; developmental stage of the C lineage expressed as total C lineage cell number. (B) DIC images of wild-type embryos representing the relative daughter cell sizes in the Caa and Caap cleavages. Length of a *C. elegans* embryo ∼50 µm. (C) Dot plot of the volumetric ratio of C lineage cleavages, expressed as the anterior/posterior (A/P) daughter volume; mean±s.d. The dashed line indicates an equal cleavage ratio of 1:1. The same colour code as in A indicates the same divisions in B and C: Caa in orange, Caap in blue. Circles on the right represent sister cell sizes at adjacent volume ratios. n.s, not significant; *P<0.5, ***P<0.001 (unpaired, two-tailed *t*-test).

The C lineage comprises two nearly bilaterally symmetric halves derived from the daughter cells Ca and Cp. Defining left-right asymmetry in the lineage, the anterior half of the lineage descended from Ca contains the neurons DVC and PVR, while the bilateral homologues descended from Cp are hypodermal (epidermal) (Fig. 1A) (Sulston et al., 1983). Like many divisions in the *C. elegans* embryo, those preceding the production of DVC and PVR are asymmetric in fate. The division of Caa represents a decision between an anterior non-neural ectodermal branch and a posterior neural/non-neural ectodermal branch (Fig. 1A). The subsequent division of Caap represents a decision between an anterior neural/cell death branch and a posterior neural and non-neural ectodermal branch, producing the DVC neuroblast and the PVR/HYP7 neural/non-neural ectodermal cells, respectively (Fig. 1A).

Through 4D-lineage analysis and volumetric quantifications, we found that these two asymmetric cell divisions are also unequal in size. Caaa is more than twice the size of Caap, and Caapp is twice that of Caapa (Fig. 1B,C). In contrast, we found that most other divisions in the C lineage exhibit either an equal cleavage or an unequal cleavage with a very small bias towards to the anterior cell (Fig. 1B,C), consistent with previous reports (Arata et al., 2014; Fickentscher and Weiss, 2017). The two most overtly unequal cleavages in the C lineage, those of Caa and Caap, differ significantly in their volume ratio to the bilaterally homologous divisions, which do not generate neurons (Fig. 1A,C). In addition to the conservation of C lineage topology in many Rhabditida species (Houthoofd et al., 2003, 2008; Memar et al., 2019; Zhao et al., 2008), we find that the unequal cleavages of Caa and Caap are also conserved over at least 20 million years of evolution in the *Caenorhabditis* genus (Fig. S1). Our lineage analysis also indicates conservation of the programmed cell death of Caapap and, based on cell position and nuclear morphology, it is highly likely that Caapaa is also a neuron in these species (Fig. S1)*.* Given such conservation, we investigated whether unequal cell size may be required for neural fate decisions in the lineage and, ultimately, DVC neuroblast specification.

### C lineage unequal cleavages are equalised in *pig-1(gm344)* and *ham-1(n1438)* mutants

**Fig. 2.**
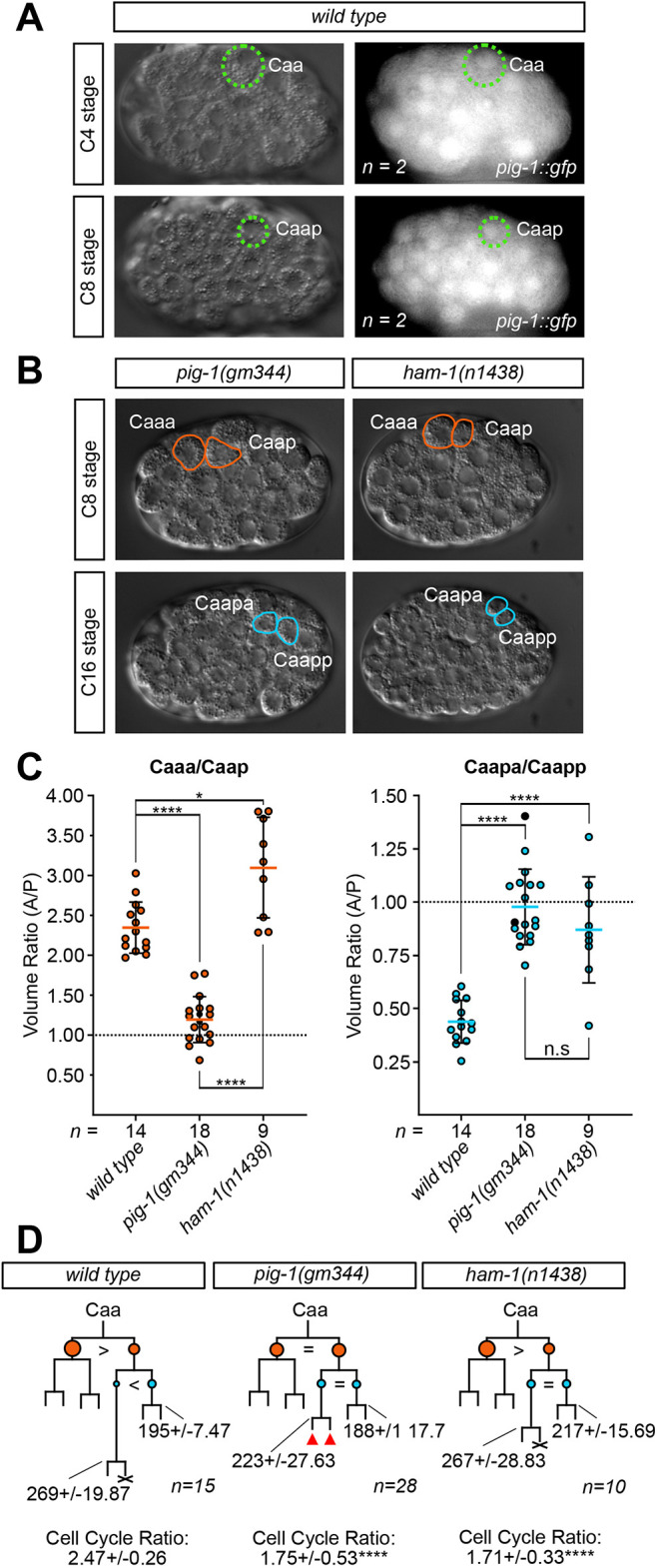
**C lineage unequal cleavages are equalised in *pig-1(gm344)* and *ham-1(n1438)* mutants.** (A) DIC and GFP images, indicating the expression of *pig-1 [bcSi43]* in wild-type embryos in Caa and Caap. Length of a *C. elegans* embryo ∼50 µm. (B) DIC images presenting the unequal cleavages of Caa and Caap in *pig-1(gm344)* and *ham-1(n1438)* embryos. (C) Dot plots of the volumetric ratio of the Caa and Caap cleavages in wild-type, *pig-1(gm344)* and *ham-1(n1438)* embryos, expressed as the anterior/posterior daughter (A/P) volume; mean±s.d. Black dots in *pig-1(gm344)* indicate no *hlh-14* expression. n.s, not significant; *P<0.05, ****P<0.0001 (one-way ANOVA with Tukey's HSD). (D) Lineage diagrams of the neurogenic branch of the C lineage in the same mutants, indicating division times and cleavage asymmetries. Data were obtained from manually 4D-lineaged embryos. Branch lengths are indicative of division times. Circles indicate the relative sizes of cells. Caa daughters indicated in orange, Caap daughters in blue. ‘=’ represents equal cleavages; ‘>’ or ‘<’ represent unequal cleavages. Red triangles indicate extra divisions sometimes seen in branch; full details in Fig. S5. X indicates cell death. Caapa and Caapp division times represented in min±s.d., with cell cycle ratio of Caapa/Caapp expression±s.d.: ****P<0.0001 (comparison with wild type, one-way ANOVA with Tukey's HSD). For details on wild-type and mutant genotypes, see Materials and Methods.

We have previously shown that the specification of DVC depends on the proneural bHLH transcription factor HLH-14 in the DVC neuroblast Caapa (Poole et al., 2011); Fig. 1A). In *hlh-14* null mutants, as well as conversion to hypodermal fate, the would-be DVC neuroblast divides precociously at the same time as the C lineage hypoblasts (Poole et al., 2011). To assess whether the unequal cleavages and resultant unequal daughter sizes are required for the expression of *hlh-14* in Caapa, we sought to equalise these cleavages. The MELK kinase PIG-1 is involved in many unequal neuroblast divisions (Cordes et al., 2006; Feng et al., 2013; Wei et al., 2020). Not previously implicated in the C lineage, we confirmed expression of *pig-1* in both Caa and Caap using a transcriptional reporter (Fig. 2A). We also found that in strong loss-of-function *pig-1(gm344)* mutants, the cleavages of both Caa and Caap are equalised (Fig. 2B,C; Fig. S2). We also observed that the DVC neuroblast Caapa divides precociously in a large proportion of lineaged embryos (Fig. 2D; Fig. S3A). To control for different developmental rates between genotypes, we also assessed this precocious division as a ratio between the cell cycle duration of Caapa and its sister Caapp. This analysis comprised additional lineaged embryos to those measured for cell volume ratio (Fig. 2D; Fig. S3B).

The STOX transcription factor HAM-1 is broadly expressed in the embryo and is also a known regulator of many unequal neuroblast divisions, particularly those that generate a smaller anterior cell (Frank et al., 2005; Guenther and Garriga, 1996; Leung et al., 2016; Teuliere et al., 2018). In the Q lineage, it is thought to act as a regulator of *pig-1* (Feng et al., 2013). In contrast to *pig-1(gm344)* mutants, in embryos carrying the loss-of-function *ham-1(n1438)* allele the volume ratio of the Caa cleavage retained an anterior bias (Fig. 2B,C). The Caap cleavage, however, demonstrated a significant reduction in volume ratio and a tendency towards equalisation (Fig. 2B,C; Fig. S2). As with *pig-1(gm344)*, a significant proportion of *ham-1(n1438)* embryos displayed a precocious Caapa division. This analysis included an additional embryo to those measured for cell volume ratio (Fig. 2D; Fig. S3). Taken together, these results indicate that *pig-1* controls unequal cleavage of both the Caa and Caap blastomeres and *ham-1* that of Caap. *pig-1* and *ham-1* mutants are therefore good genetic backgrounds in which to assess the role of these unequal cleavages on the acquisition of asymmetric daughter cell fates, particularly the asymmetric expression of *hlh-14* in Caapa.

### Equalisation of the Caa or Caap blastomere cleavages in *pig-1* and *ham-1* mutants has little effect on *hlh-14* expression in the DVC neuroblast Caapa

**Fig. 3.**
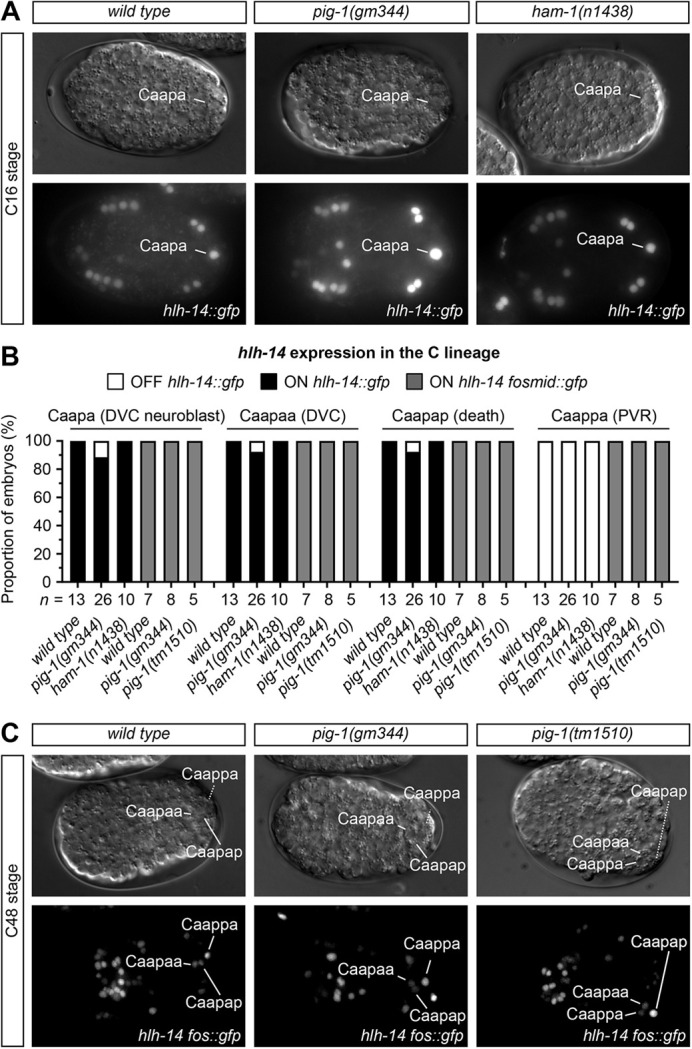
***hlh-14* expression in *pig-1(gm344)*, *pig-1(tm1510)* and *ham-1(n1438)* mutants.** (A) DIC images and GFP maximum intensity projections for *hlh-14* expression *[gmIs20]* in wild-type, *pig-1(gm344)* and *ham-1(n1438)* bean-stage (C16) embryos. Lines indicate expression in the DVC neuroblast Caapa. Length of a *C. elegans* embryo ∼50 µm. (B) Stacked bar chart of expression of *hlh-14 [gmIs20]* in the Caapa, Caapaa, Caapap and Caappa in wild-type, *pig-1(gm344)* and *ham-1(n1438)*, and of *hlh-14 [hlh-14 fosmid::gfp]* expression in wild-type, *pig-1(gm344)* and *pig-1(tm1510)* lineaged embryos. Wild-type fates of cells indicated in brackets. (C) DIC and GFP images of *hlh-14* expression *[hlh-14 fosmid::gfp]* in *pig-1(gm344)* and *pig-1(tm1510)* late bean-stage (C48) embryos following terminal C lineage divisions. Representative of majority phenotypes. For details on wild-type and mutant genotypes, see Materials and Methods.

4D-lineage analysis using a translational fusion reporter revealed that the equalisation of the Caap blastomere cleavage in *ham-1(n1438)* embryos has no effect on the expression of *hlh-14. hlh-14* was detected in Caapa and, after division, in its daughters Caapaa (DVC) and Caapap (death) in 10/10 lineaged embryos, which includes those nine measured in Fig. 2 (Fig. 3A,B). Similarly, despite equalisation of both cleavages in *pig-1(gm344)* embryos, *hlh-14* was expressed in the DVC neuroblast Caapa in 23/26 lineaged embryos in which expression could be analysed, including all those measured for Caa and Caap in Fig. 2. Following division, 24/26 continued to express *hlh-14* in Caapaa and Caapap (Fig. 3A,B). Furthermore, transgene expression was never evident in an ectopic branch. For example, equalisation of the Caa cleavage did not result in ectopic expression in Caaa or any of its descendants (Figs S5 and S6).

**Fig. 4.**
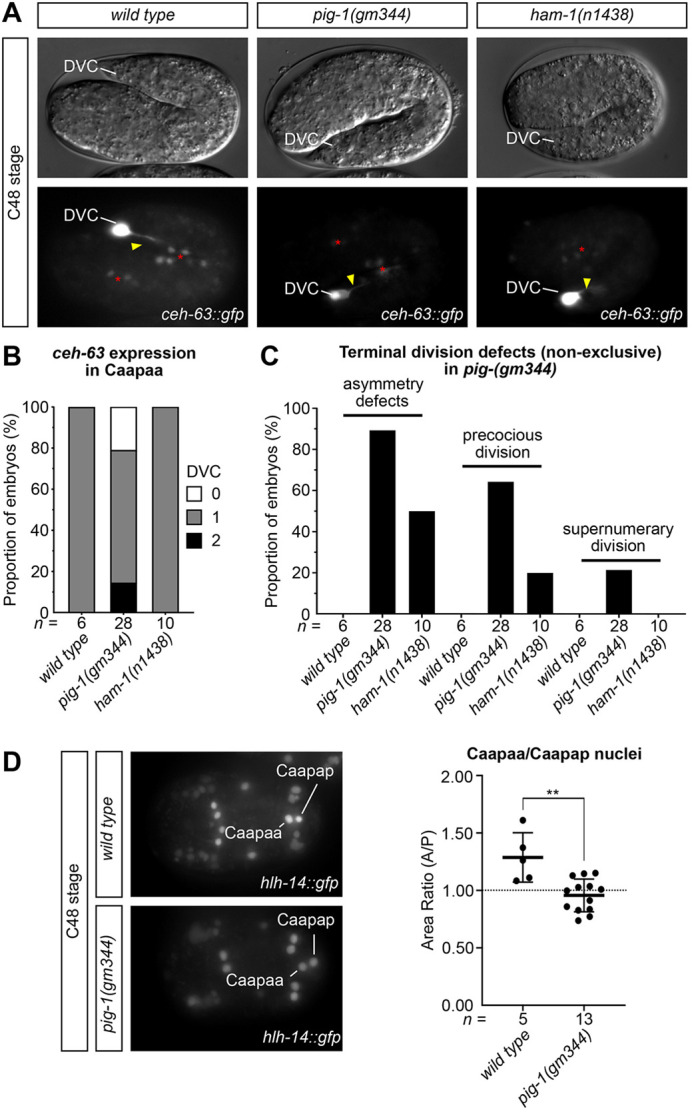
**Terminal division defects in *pig-1(gm344)* and *ham-1(n1438)*.** (A) DIC and GFP images for *ceh-63 [otIs458]* expression in wild-type, *pig-1(gm344)* and *ham-1(n1438)* 2-fold stage (C48) embryos. DVC neuron is indicated. Yellow arrowheads indicate neuronal projections, red asterisks indicate residual *hlh-14* expression*.* Length of a *C. elegans* embryo ∼50 µm. (B) Stacked bar chart representing the number of *ceh-63 [otIs458]*-expressing cells at elongation stage in lineaged *pig-1(gm344)* and *ham-1(n1438)* embryos. (C) Bar chart representing terminal division phenotypes in lineaged *pig-1(gm344)* and *ham-1(n1438)* embryos. Categories are non-exclusive. Asymmetry defects, all non-wild-type phenotypes; precocious division, precocious division of Caapa; supernumerary division, extra rounds of division of any Caapa daughter cells. (D) Left: GFP maximum intensity projection images for *hlh-14 [gmIs20]* expression in lineaged wild-type and *pig-1(gm344)* embryos following Caapa division, indicating a loss of size asymmetry in *pig-1(gm344)* mutants. Right: Dot plot of nuclear area ratio of the Caapa daughters in wild-type and *pig-1(gm344)* embryos, expressed as the anterior/posterior (A/P) daughter area; mean±s.d. **P<0.01 (unpaired, two-tailed *t*-test). For details on wild-type and mutant genotypes, see Materials and Methods.

Assessment of *hlh-14* expression was also conducted using another fosmid-based translational fusion reporter. As in wild-type embryos, both *pig-1(gm344)* and another strong loss-of-function allele, *pig-1(tm1510)*, displayed *hlh-14* expression in the DVC neuroblast and its descendants in all lineaged embryos (Fig. 3B,C). In addition to expressing *hlh-14*, the position of the DVC neuroblast Caapa in *pig-1* and *ham-1* resembled that observed in wild-type embryos, occupying a central position in the posterior of the embryo during the C16 stage (Fig. 4A). This is in contrast to *hlh-14* mutants, in which Caapa migrates to a lateral position at the same stage, adjacent to hypodermal cells, itself having adopted a hypodermal fate (Poole et al., 2011). Unlike the translational reporter, the fosmid-based reporter is also expressed in the PVR neuron. We found that *hlh-14* was expressed in Caappa (PVR) in all lineaged *pig-1* mutant embryos (Fig. 3B,C). Taken together, these results suggest that the unequal cleavages of the Caa and Caap blastomeres and resultant smaller daughter cell sizes are not the main factor determining asymmetric expression of *hlh-14* in the DVC neuroblast (Caaapa) and the PVR neuron.

### *pig-1* and *ham-1* affect the asymmetric division of the DVC neuroblast

We next investigated whether cleavage equalisation affects later steps in C lineage neurogenesis downstream of *hlh-14* expression. Specifically, we examined whether the Caapaa blastomere still acquires DVC fate. This was assessed in the same embryos using a transcriptional fusion reporter for the DVC-specific transcription factor *ceh-63* (Feng et al., 2012). Although *hlh-14* and *ceh-63* expression were both assessed with GFP transgenes in the same animal, they could be assessed simultaneously in the same 4D-lineaged embryo as they are expressed at different embryonic stages in a non-overlapping manner and because the *hlh-14::gfp [gmIs20]* reporter is nuclear whereas the *ceh-63::gfp* reporter is cytoplasmic. A further two *pig-1(gm344)* mutant embryos in addition to the 26 lineaged for *hlh-14* expression (Fig. 3) were assessed for *ceh-63* expression. The majority displayed a single DVC neuron; 6/28 embryos lacked a neuron and 4/28 displayed two neurons. (Fig. 4A,B). In hatched L1 *pig-1(gm344)* larvae, both single and ectopic DVC neurons displayed a normal morphology and displayed neuronal processes (Fig. S4). As with the equalisation phenotypes, these phenotypes were weaker in *ham-1(n1438)* mutants in which all embryos displayed a single neuron (Fig. 4A,B).

Lineage analysis revealed that *pig-1(gm344)* mutants display a range of terminal DVC neuroblast division defects akin to those described in other lineages (Cordes et al., 2006; Ou et al., 2010; Feng et al., 2013; Zhu et al., 2014; Wei et al., 2017). Only 3/28 lineaged embryos displayed a wild-type fate pattern at the terminal division (Fig. S5). The remaining 25/28 embryos displayed an asymmetry defect at the terminal division, with the inappropriate survival of Caapap, and subsequent adoption of DVC fate by one, both or neither of Caapaa and Caapap (Fig. 4C; Fig. S5). A precocious division of Caapa (the DVC neuroblast) was observed in 18/25 of these embryos. In 6/18 of those embryos, Caapa daughter cells underwent an extra round of division (Fig. 4C; Fig. S5). This resulted in supernumerary *hlh-14*-expressing cells and, in some cases extra DVC neurons (Fig. S5). Extra divisions were confined to the Caapa branch with the supernumerary DVCs arising from either the inappropriately surviving DVC sister cell or the supernumerary DVC neuroblast descendants. Only 4/28 embryos displayed a clear cell death in any cell by the end of the recording, including Caapap, which is normally fated to die (Fig. S5). All lineaged *ham-1(n1438)* embryos displayed a single DVC neuron with Caapaa correctly adopting DVC fate in 8/10 embryos, and Caapap doing so in 2/10. However, only 4/10 embryos displayed a wild-type terminal division pattern in which Caapa does not divide precociously, Caapaa adopts DVC fate and Caapap undergoes apoptosis. Overall, the loss of apoptosis was also milder in *ham-1(n1438)* embryos with a cell death evident in 7/10 embryos (Fig. S6).

Having seen this effect on terminal fates, and the equalisation of preceding cleavages, we wondered whether the terminal division of Caapaa is also equalised in *pig-1(gm344)* mutants. Cell size at the terminal division provided a technical challenge to DIC image measurement. However, as nucleus size can be used as a proxy for overall cell size (Ginzberg et al., 2018), we took advantage of the fact that the *hlh-14::gfp [gmIs20]* reporter is both nuclear and expressed in *pig-1(gm344)* mutants. We found that the nuclear size ratio at the Caapaa cleavage displayed an anterior bias in wild-type embryos and that Caapap, fated to die, is smaller (Fig. 4D). This was significantly equalised in *pig-1(gm344)* mutants (Fig. 4D). Despite the DVC neuroblast itself being significantly enlarged owing to the equalisation of the Caa and Caap divisions, the equalisation of the Caapa cleavage resulted in a similarly sized nucleus in *pig-1(gm344)* mutants and wild types (Figs S2 and S7). Combined, these lineage and terminal division size ratio analyses demonstrate that both *pig-1* and *ham-1* play a role in the both the unequal cleavage of the DVC neuroblast and the correct acquisition of asymmetric terminal cell fates (neuronal fate versus cell death).

### The Caap blastomere cleavage is affected in *hlh-14(tm295)* mutants

**Fig. 5.**
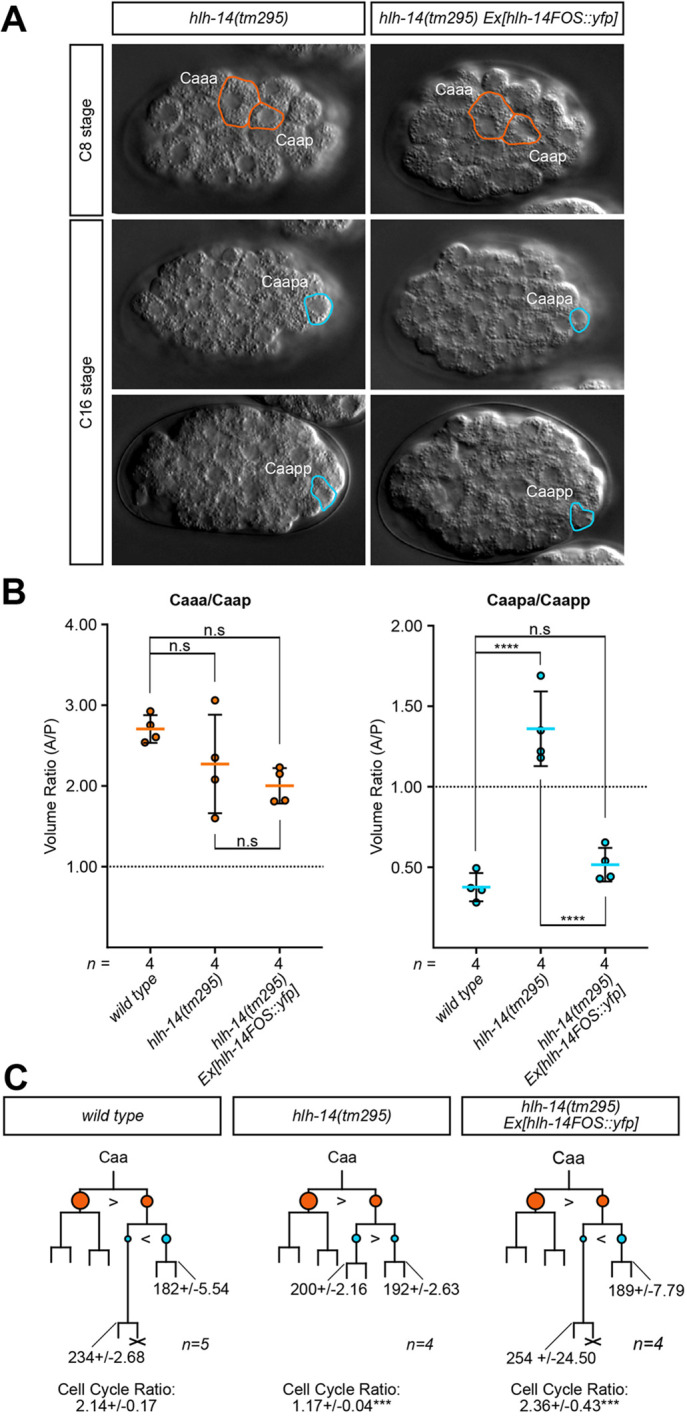
**The Caap blastomere cleavage is affected in *hlh-14(tm295)* mutants.** (A) DIC images of *hlh-14(tm295)* and *hlh-14 fosmid*-rescued embryos representing the relative daughter cell sizes at the Caa and Caap cleavages. Length of a *C. elegans* embryo ∼50 µm. (B) Dot plots of the volumetric ratio of the Caa and Caap cleavages in wild-type and *hlh-14(tm295)* embryos, expressed as the anterior/posterior (A/P) daughter volume; mean±s.d. n.s, not significant; ****P<0.0001 (one-way ANOVA with Tukey's HSD). Dashed line indicates absolute equal cleavage ratio of 1:1. (C) Lineage diagrams of the neurogenic branch of the C lineage for wild-type, *hlh-14(tm295)* and *hlh-14(tm295) fosmid*-rescued embryos, constructed from manually 4D-lineaged embryos. Branch lengths are indicative of division timings. Circles represent relative sizes of cells; with ‘>’ or ‘<’ representing the larger cell. Orange indicates Caa daughters, blue Caap daughters. X indicates cell death. Caapa and Caapp division times represented in min±s.d., with cell cycle ratio of Caapa/Caapp expression±s.d. ***P<0.001 (comparison with wild type, one-way ANOVA with Tukey's HSD). For details on wild-type and mutant genotypes, see Materials and Methods.

The disruption of the unequal cleavages of Caa and Caap in *pig-1(gm344)* and *ham-1(n1438)* mutants has minimal impact on the expression of *hlh-14*. We therefore wondered what the purpose of these unequal cleavages may be and how they are regulated. We hypothesised that, given the lack of embryonic cell growth, these unequal cleavages may be regulated to control terminal cell size and speculated that they may therefore be under the control of the same factors that regulate cell fate acquisition. 4D-lineage analysis of *hlh-14(tm295)* null mutants revealed no defect in the Caa cleavage. In contrast, the unequal cleavage of Caap was strongly affected and resembled the hypoblast/hypoblast divisions of other C lineage branches (Fig. 5A,B; Fig. S2). Also, in agreement with previous work (Poole et al., 2011), 4D-lineaging confirmed that Caapa divides precociously in *hlh-14(tm295)* (Fig. 5C; Fig. S3). These results suggest that the proneural transcription factor HLH-14 acts to regulate the unequal cleavage and resultant daughter size asymmetry at the Caap division. In contrast, detected expression of the *hlh-14* transgenes only began in the daughter cells themselves. However, close analysis of an *hlh-14 fosmid::gfp* transgene pattern and timing demonstrated two distinct phases of expression with the first detected as soon as 11 min post-division (Fig. S8). Given the maturation time and photostability of GFP (Balleza et al., 2018; Heppert et al., 2016), this early phase of expression is consistent with transcription of *hlh-14* in Caap, the mother of Caapa and Caapa.

### *let-19* is an upstream regulator of *hlh-14* in the C lineage

Reasoning that upstream regulators of *hlh-14* would exhibit a precocious division of the DVC neuroblast Caapa, we performed a 4D lineage-based screen of temperature-sensitive, embryonic-lethal mutants for such a phenotype. This screen revealed a mutant allele, *t3273*, which mapping-by-sequencing, complementation testing and rescue experiments revealed is an allele of the Mediator complex kinase module subunit LET-19 (Fig. S9; see Materials and Methods). Many of the Mediator complex subunits are ubiquitously expressed in *C. elegans* (Steimel et al., 2013; Wang et al., 2004; Zhang and Emmons, 2000) and we confirmed *let-19* expression in Caa, Caap and their descendants (Fig. S10).

**Fig. 6.**
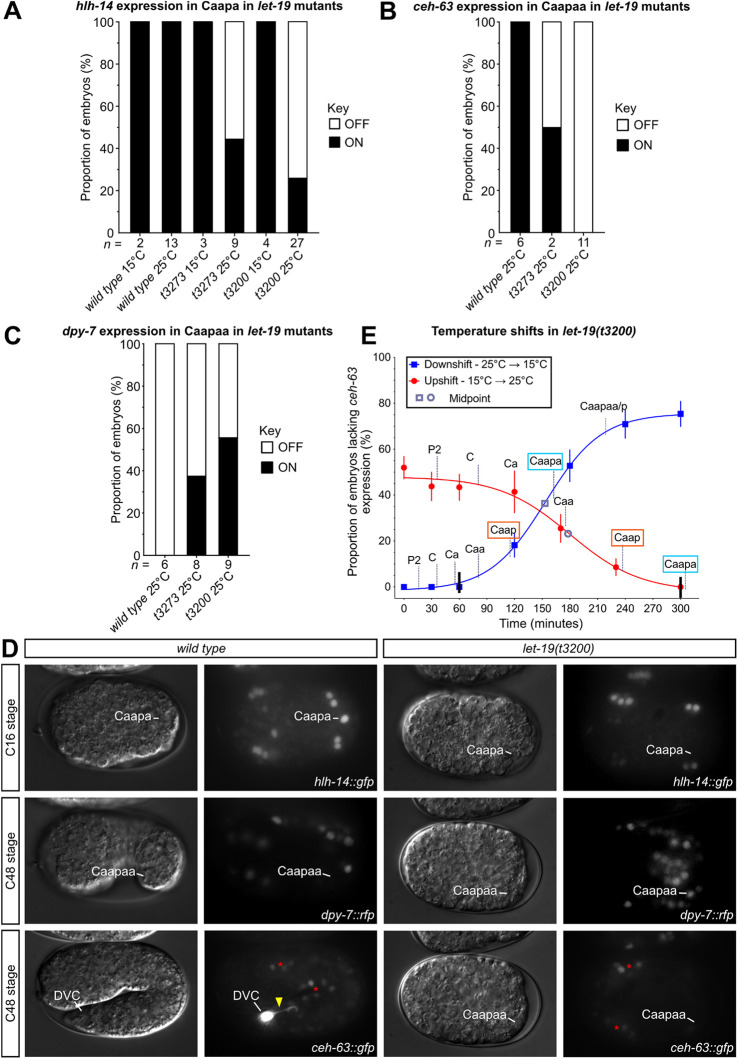
**Loss of *hlh-14* expression and adoption of hypodermal fate in *let-19* mutants.** (A) Stacked bar chart for *hlh-14 [gmIs20]* expression in Caapa (DVC neuroblast) in lineaged wild-type, *let-19(t3273)* and *let-19(t3200)* embryos at the permissive temperature 15°C and non-permissive temperature 25°C. Wild-type and *let-19(t3200)* embryos include embryos carrying only the *hlh-14 [gmIs20]* transgene or *hlh-14 [gmIs20]*, *dpy-7 [stIs10166]* and *ceh-63 [otIs458]* transgenes. *let-19(t3273)* embryos carried only *hlh-14 [gmIs20]*. (B) Stacked bar chart for *ceh-63 [otIs458]* expression in Caapaa in lineaged wild-type and *let-19* mutant embryos, at non-permissive temperature 25°C. Wild-type and *let-19(t3200)* embryos include embryos carrying *hlh-14 [gmIs20]*, *dpy-7 [stIs10166]* and *ceh-63 [otIs458]* transgenes. *let-19(t3273)* embryos carried only *ceh-63 [otIs458].* (C) Stacked bar chart for *dpy-7 [stIs10166]* expression in Caapaa in lineaged wild-type and *let-19* mutant embryos, at non-permissive temperature 25°C. Wild-type and *let-19(t3200)* embryos include embryos carrying *hlh-14 [gmIs20]*, *dpy-7 [stIs10166]* and *ceh-63 [otIs458]* transgenes. *let-19(t3273)* embryos carried only *dpy-7 [stIs10166].* (D) DIC and GFP maximum intensity projection images of *hlh-14* expression *[gmIs20]* in bean-stage (C16), RFP maximum intensity images of *dpy-7* expression *[stIs10166]* in comma-stage (C48) and GFP maximum intensity projection images of *ceh-63* expression *[otIs458]* in twofold (C48)-stage wild-type and *let-19(t3200)* embryos carrying all three reporters. Mutant phenotypes are represented. Positions of the Caapa (DVC neuroblast) or Caapaa (DVC in wild type) are indicated. Red asterisks indicate residual *hlh-14* expression in *ceh-63* images and yellow arrowhead indicates neuronal projection. Length of a *C. elegans* embryo ∼50 µm. (E) Percentage loss of *ceh-63* expression (DVC) curves in *let-19(t3200)* for upshifts from permissive to non-permissive temperature (15°C→25°C) are plotted in red with downshifts in blue (25°C→15°C). Black lines indicate the window of critical time point for LET-19 action. A grey circle or square indicates the mid-point of up- and downshift curves, respectively. Cell names are represented at birth times at each temperature condition on the relevant curve. *x*-axis represents time points for the temperature shift. The birth of Caap is indicated by an orange box, Caapa in blue. *n=*40-60 for each time point in the downshift experiments (except *n=9* at 0 min). *n=*50-70 per time point for upshifts (except *n=*102 at 0 min, *n=*29 at 120 min).

Following identification, we characterised *let-19(t3273)* mutants together with a second *let-19* allele, *t3200*, and assessed whether they shared additional phenotypes with *hlh-14* mutants, namely loss of both *hlh-14* expression and neuronal cell fates (Poole et al., 2011). In gastrulation stage *let-19(t3273)* mutants, *hlh-14* expression was lost in the would-be DVC neuroblast Caapa in just over half of the lineaged embryos at the non-permissive temperature of 25°C, with a far higher penetrance observed in *let-19(t3200)* (Fig. 6A). Downstream of *hlh-14* expression, DVC cell fate was assessed using the transcriptional reporter for the unique DVC marker *ceh-63. let-19(t3200)* displayed a higher penetrance of loss with all embryos lacking *ceh-63* expression (Fig. 6B). A histone-RFP fusion driven by the promoter of the hypodermal marker *dpy-7* was used to assess adoption of hypodermal (non-neural ectodermal) fate with *let-19(t3200)* again displaying a more highly penetrant phenotype. For both alleles, it is unclear why the adoption of hypodermal fate in Caapaa displays a lower penetrance than that of the loss of the *hlh-14* expression. For *let-19(t3200)*, a subset of the embryos analysed carried all three reporters at once, which allowed concomitant assessment in the same embryo. In these embryos, *ceh-63* expression was never detected in embryos lacking *hlh-14.* Conversely, *dpy-7* was only detected in those embryos lacking *hlh-14* expression (Fig. 6D). We also observed that *let-19* mutant animals display a very high degree of embryonic lethality (Fig. S9) and frequently fail to undergo morphogenesis. It is notable that, in addition to a loss of *hlh-14* expression, Caapa occupies a lateral position in the posterior of the embryo at the C16 stage, contrasting the central position of the wild-type cell (Fig. 6D). This is also the position of Caapa in *hlh-14* null mutants (Poole et al., 2011), further supporting the loss of neuronal cell fate and transformation to a hypoblast. Together, these observations demonstrate that *let-19* is required for the correct specification of the DVC neuroblast.

### *let-19* regulates the unequal cleavage of the Caa, and possibly Caap, blastomeres

**Fig. 7.**
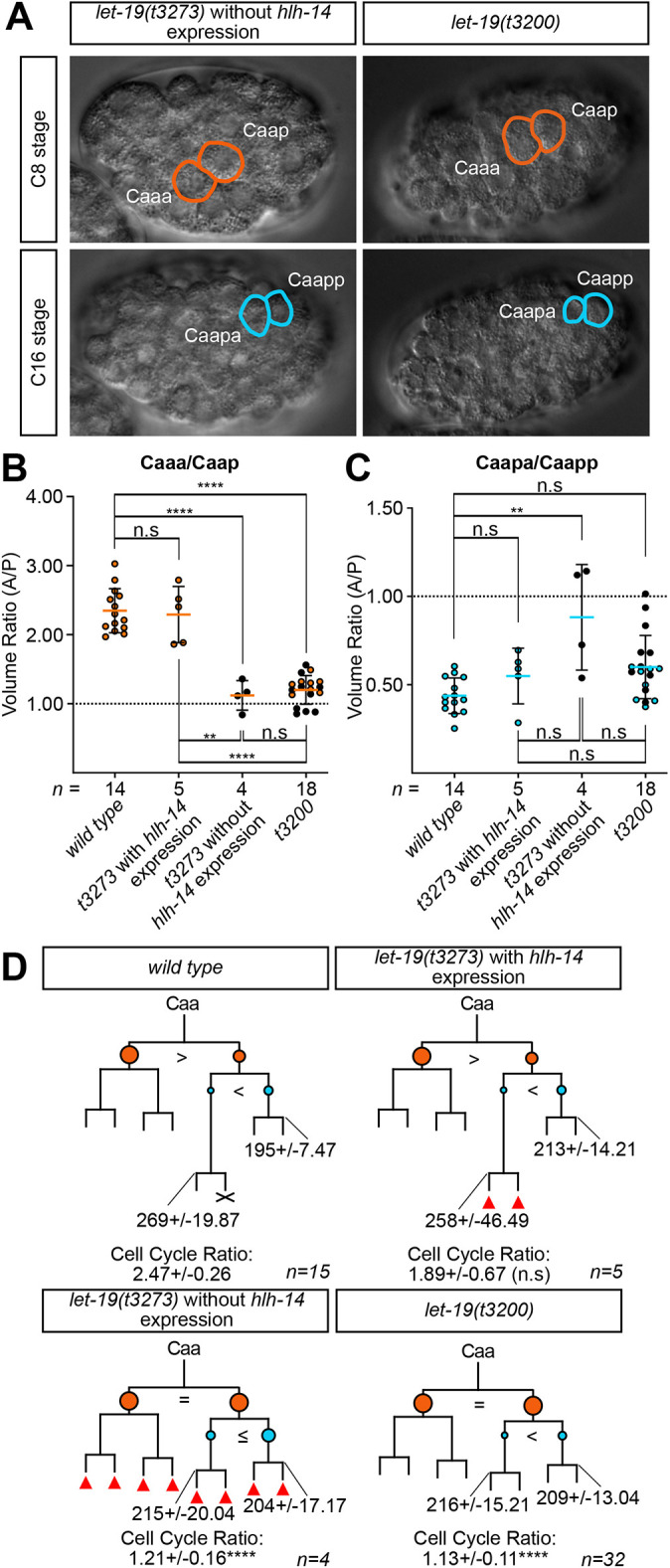
***let-19* regulates the unequal cleavage of the Caa and possibly Caap blastomeres.** (A) Representative DIC images of *let-19(t3200)* and *let-19(t3273)* mutants lacking *hlh-14* expression, representing relative daughter cell sizes at the Caa and Caap cleavages. (B) Dot plot of the volumetric ratio of the Caa cleavage in wild type and *let-19* mutants, expressed as the anterior/posterior (A/P) daughter volume; mean±s.d. For *let-19(t3273)*, the proportion of embryos that express *hlh-14* are plotted separately from those that do not. *let-19(t3200)* embryos lacking *hlh-14 [gmIs20]* expression are depicted as black. Dashed line indicates an absolute equal cleavage ratio of 1:1. n.s, not significant; **P<0.01, ****P<0.0001 (one-way ANOVA with Tukey's HSD). (C) The same as for B but for the Caap cleavage. *let-19(t3200)* embryos lacking *hlh-14 [gmIs20]* expression are depicted in black. **P<0.01 (one-way ANOVA with Tukeys's HSD). (D) Lineage diagrams of the neurogenic branch of the C lineage in *let-19* mutants indicating division times and cleavage asymmetries. Data were obtained from manually 4D-lineaged embryos. Branch lengths indicative of division timings. Circles represent relative cell sizes; ‘=’ represents an equal cleavage; ‘>’ or ‘<’ indicate the larger cell. Orange indicates Caa daughters, blue Caap daughters. Red triangles indicate extra divisions and X indicates cell death. Caapa and Caapp division times represented in min±s.d., with cell-cycle ratio of Caapa/Caapp expression±s.d.; n.s, not significant; ****P<0.0001 (comparison with wild type, one-way ANOVA with Tukey's HSD). For details on wild-type and mutant genotypes, see Materials and Methods.

Taking advantage of the temperature-sensitive nature of the *let-19* alleles, we aimed to define the timing of action for LET-19 in the regulation of *hlh-14* expression and neurogenesis in the lineage. As the stronger and more consistent allele, assessment of this critical period was undertaken with *let-19(t3200).* Timed temperature shifts from both permissive to non-permissive (15°C to 25°C) temperatures and non-permissive to permissive (25°C to 15°C) and assessment of the loss of *ceh-63* expression in Caapaa (DVC) established that LET-19 acts around the time of the Caa cleavage (Fig. 6E). We found that in all *let-19(t3200)* embryos analysed, there is a striking equalisation of this cleavage (Fig. 7A,B). To quantify this equalisation, a subset of the *let-19(t3200)* embryos assessed for *hlh-14* expression as shown in Fig. 6 were measured for cell volumes and all displayed a significant equalisation of the Caa cleavage ratio (Fig. 7B; Fig. S2A). Of the nine *let-19(t3273)* embryos scored for *hlh-14* expression in Caapa in Fig. 6, seven were measured for cell volumes. A further two embryos in which *hlh-14* expression was assessed in Caapaa instead (not included in Fig. 6), were also measured. This allowed the concordance between the presence or absence of *hlh-14* expression in the C lineage and cell volume ratios to be assessed in this second set of nine embryos. We found that the Caa cleavage ratio is significantly equalised in those embryos lacking *hlh-14* expression (Fig. 7B). We also observed defects in the unequal cleavage of Caap, particularly in *let-19(t3273)* embryos lacking *hlh-14* expression, which also showed a significant equalisation of the cleavage. This was milder in *let-19(t3200)* mutants, in which the cleavage mostly resembled the wild type (Fig. 7C).

We also quantified the division time of Caapa in both *let-19* mutants. Precocious division of Caapa was observed in all *let-19(t3200)* mutants regardless of *hlh-14* expression (Fig. 7D; Fig. S3)*.* As with the cell volume ratio defects, precocious division of Caapa correlated with *hlh-14* expression in *let-19(t3273)*, with those lacking *hlh-14* demonstrating a precocious Caapa division (Fig. 7D; Fig. S3). In addition, a highly variable extra division phenotype was observed in various branches of the C lineage affecting 3/9 *let-19(t3273)* embryos, with 1/5 *let-19(t3273)* of those expressing *hlh-14* and 2/4 lacking *hlh-14* expression (Fig. 7D). In addition to its role as an upstream regulator of *hlh-14* expression, and therefore the acquisition of neural fate, these results suggest that *let-19* strongly regulates the unequal cleavage of Caa, and more weakly that of Caap, thereby controlling the size of their daughters.

## DISCUSSION

How cell size is specified during development, how cells sense size and the consequences of cell size on cell fate and function are fundamental questions in biology. Cell size can be regulated by either cell growth or the total number of divisions. It can also be more specifically regulated by the control of daughter cell size asymmetry during mitosis. Many asymmetric divisions that generate daughter cells of different fates are also unequal, generating daughter cells of different sizes. How and why this occurs during development is unclear. One possibility is that unequal cell size directly affects asymmetric cell fate decisions. A clear example of this is seen in the alga *Volvox carteri*, in which cleavage plane manipulation experiments result in daughter cell fate defects (Kirk et al., 1993). However, in other contexts, such as exit of pluripotency in the daughters of asymmetric embryonic stem cell divisions, cell size does not appear to play any role in cell fate decisions (Chaigne et al., 2020). The work presented in this study provides evidence that cell size alone, through unequal cleavage, does not play a major role in the expression of the proneural gene *hlh-14/ASCL1* and so the specification of DVC neuroblast fate. We can therefore conclude that in a variety of different contexts cell size does not affect certain aspects of cell fate specification.

We have shown here that *pig-1* regulates the unequal cleavage of Caa and Caap, and that Caap cleavage is also regulated by *ham-1*. These genes have been previously described to regulate the unequal cleavages of asymmetrically dividing *C. elegans* neuroblasts with a smaller daughter that dies, such as in the Q and NSM neuroblast (Cordes et al., 2006; Feng et al., 2013; Frank et al., 2005; Guenther and Garriga, 1996; Leung et al., 2016; Wei et al., 2017; Teuliere et al., 2018). Although *pig-1* has a redundant role in the unequal division of the 1-cell embryo and the EMS blastomere at the 4-cell stage (Liro et al., 2018; Morton et al., 2012; Pacquelet et al., 2015), to our knowledge this is the first example of it regulating successive divisions in a lineage. This is likely through one of two mechanisms employed in other *C. elegans* cleavages: asymmetric spindle positioning or control of cortical myosin distribution and contractility (Ou et al., 2010; Wei et al., 2020).

We observed little effect on the expression of *hlh-14* in the DVC neuroblast as a result of equalising either the Caa or Caap divisions in *pig-1* or *ham-1* mutants. Nor was *hlh-14* detected ectopically in a sister branch of an equalised cleavage. Indeed, the production of a DVC neuron was also not abolished in most *pig-1* or *ham-1* mutant embryos. Together, these observations suggest that equalisation does not prevent correct DVC neuroblast specification and, as such, neural fate specification in this lineage. However, *pig-1(gm344)* clearly does affect the terminal division of the DVC neuroblast in a number of ways. As in previously investigated lineages, we found that PIG-1 regulates the unequal cleavage of the DVC neuroblast and disrupts the segregation of neuronal and apoptotic fate. This is evidenced by the reversal of the division and/or the inappropriate survival of the DVC sister cell normally fated to die and therefore the loss or duplication of DVC. Furthermore, *pig-1* mutants also displayed cell cycle defects, such as the precocious and supernumerary divisions of the DVC neuroblast, providing a source of supernumerary DVC neurons. One intriguing possibility for future investigation is that the DVC blastomere may somehow sense that it is too large. This could then either lead it to divide early, consistent with the power-law relationship previously described between cell size and cell cycle timing (Arata et al., 2014), or to even undergo an extra round of division as we observed in a small fraction of *pig-1* mutants. Consistent with this possibility, we observed that in *pig-1* mutants, the larger the DVC neuroblast is the more likely it is to undergo an extra division (Fig. S7).

Through a forward genetic screen we have identified the Mediator complex kinase module component LET-19 as a regulator of neural specification in the C lineage. In *let-19* mutants, *hlh-14* expression was lost, the DVC neuroblast divided precociously and its daughters acquired a hypodermal cell fate. The Mediator complex is an evolutionarily conserved regulator of transcriptional events, including those in *C. elegans* (Grants et al., 2015), and as our mutants are embryonic-lethal, C lineage neurogenesis is likely one of many molecular events affected by these alleles. However, their temperature-sensitive nature permitted identification of the critical period of action LET-19 in our case, around the time of the Caa division. Indeed, in addition to *let-19*, the three other kinase module components *dpy-22/MDT12*, *cdk-8/CDK8* and *cic-1/CCNC(Cyclin C)*, have been implicated in specific cells during neuronal development in the worm (Doitsidou et al., 2018; Luo and Horvitz, 2017; Wang et al., 2004; Zhang and Emmons, 2000) and in asymmetric cell divisions more generally (Grants et al., 2016; Yoda et al., 2005). What is perhaps surprising is that we show here that *let-19* is also required for the unequal cleavage of Caa. This is to our knowledge the first account of *let-19* affecting an unequal cleavage. Furthermore, we find that *hlh-14* regulates the unequal cleavage of Caap, in addition to specifying DVC neuroblast cell fate. As an upstream regulator of *hlh-14*, why do *let-19* mutants not phenocopy *hlh-14* mutants in terms of cleavage equalisation rather than affecting different cleavages? An explanation possibly lies in the two distinct phases of *hlh-14* expression in Caapa that we observed (Fig. S8). The early phase is consistent with transcription of *hlh-14* in Caap, appearing as early as 11 min post-division; LET-19 may only be required to regulate the second later phase of *hlh-14* in Caapa. Altogether, our results allow us to conclude that two successive unequal divisions in the C lineage are regulated by key neural cell fate regulators to determine embryonic neuroblast cell size. This parallel regulation of the cellular (cell size) and molecular (neuronal fate) aspects has been described in *C. elegans* before. In the Q lineage, the proneural factor *lin-32/ATONAL* affects both unequal cleavages and fate acquisition (Zhu et al., 2014). In this context, our results present a clear second example of proneural gene dependent regulation of cell size and may therefore indicate a conserved principle in *C. elegans* in the regulation of cell size and fate acquisition in tandem by the same factors.

From a developmental perspective, the fact that the *C. elegans* embryo lacks cell growth and has an invariant cell lineage and a fixed number of cells puts potential constraints on the size of cells generated by equal cell cleavages (Sulston et al., 1983). With this lack of growth, the spatial arrangement and size of cells may require tight regulation to produce intact, functional tissues as the worm hatches. Recently, this has been described in the *Ciona* embryonic tailbud, where unequal cleavages producing cells of symmetric fate are required for correct morphogenesis (Winkley et al., 2019). From a functional perspective, cell types have defined sizes and morphologies linked to function (Ginzberg et al., 2015). The size of neuronal cell bodies is linked to neuronal function owing to its impact on ion channel density and action potential strength and efficiency (Sengupta et al., 2013). Neurons have the smallest soma of all *C. elegans* cells, born only after the 10th or 11th cleavage round in the AB or MS lineages (Sulston and Horvitz, 1977; Sulston et al., 1983). The C lineage produces large hypodermal cells and the small DVC neuron after the 8th cleavage. In the absence of general cell growth or shrinking in the embryo, two unequal divisions appear to be an efficient solution for scaling cell size down to a small neuron. If this were the case one might expect that control of these unequal cleavages is linked to the acquisition of cell fate and this is exactly what we observe.

We have shown that, in addition to the overall topology of the C lineage, the two unequal cleavages of Caa and Caap are conserved over 20 million years of *Caenorhabditis* evolution (this study; Zhao et al., 2008; Memar et al., 2019). It has been argued that the higher developmental rates observed in *Caenorhabditis* species produced an evolutionary pressure towards generating cells in the correct position in the embryo, rather than relying on extensive cell migrations (Houthoofd et al., 2003). In support of this, there is a greater degree of lineage monoclonality in more basal species (Schulze and Schierenberg, 2011, 2009; Schulze et al., 2012). Interestingly, although the C lineage is highly conserved in the distantly related Rhabditids *Pellioditis marina* and *Rhabditophanes sp.*, in the slower developing species *Halicephalobus gingivalis* it produces only hypodermal and muscle cells (Houthoofd et al., 2003, 2008; Houthoofd and Borgonie, 2007). The homologous neurons appear to be generated from the AB lineage, migrating to their final position in the posterior of the embryo (Houthoofd et al., 2003). It is therefore tempting to speculate that the constraints of rapid developmental timing and an invariant lineage that produces polyclonal neural fate specification may combine with the functional requirement for neurons to be small discussed above. This could therefore necessitate co-regulation of unequal cleavages and neuronal fate to efficiently control fate and cell size in a branch that only undergoes eight cell cycles. Together, our results lead to the proposal that the fate regulator-dependent control of cell size via unequal cleavage is a versatile molecular mechanism to generate cell size differences in the absence of cell growth and volume modulation.

## MATERIALS AND METHODS

### *C. elegans* strain procurement and maintenance

*C. elegans* strains used in this study were derived from the N2 reference strain and were maintained at 20°C, with temperature-sensitive strains maintained at 15°C in accordance with standard practice (Stiernagle, 2006). A number of strains were obtained from the *Caenorhabditis* Genetics Center, based at the University of Montana, USA (https://cgc.umn.edu/). NG4280 was obtained from the National Bioresource Project for *C. elegans* housed at the Mitani Lab at the Tokyo Women's Medical University School of Medicine, Japan (https://shigen.nig.ac.jp/c.elegans/). Some strains were kind gifts from Oliver Hobert (Columbia University, New York, USA) and Barbara Conradt (University College London, UK). See Table S1 for a full list of strains used.

### Microscopy and imaging

DIC (Nomarski) and fluorescence imaging was undertaken with a Zeiss Axio ImagerM.2 upright microscope (Carl Zeiss) using a 100×/1.3 oil immersion objective and mounted pco.sensicam or pco.edge 3.1 (PCO) sCMOS camera. For fluorescence imaging, illumination was controlled by either a Colibri.2 (Carl Zeiss) or Cool LED pE-2 (Cool LED) LED system. Image acquisition was controlled using the bespoke software TimeToLive (Caenotec, Prof. Ralf Schnabel, Börssum, Germany). For DIC and fluorescent imaging, 4D-lineage recording and phenotypic scoring embryos were mounted in the same manner. One- or two-cell embryos were collected by bisecting gravid hermaphrodites suspended in a drop of M9 buffer with a scalpel blade. Molten 2% agarose was flattened between glass microscope slides and the embryos placed on the resultant pad via mouth pipette. A coverslip was gently placed over the pad and M9 buffer introduced under to the pad for hydration. Melted petroleum jelly was used to seal the coverslip to the slide.

### 4D-lineaging

4D-lineaging was performed as described by Schnabel et al. (1997). Image acquisition for 4D-lineaging was achieved using a Zeiss Axio Imager.M2 microscope mounted with a pco.edge 3.1 sCMOS camera or a pco.sensicam (PCO Kelheim, Germany). Recordings at the non-permissive temperature of 25°C consisted of 750 DIC *z*-stacks acquired at 35 s intervals with 25 slices per stack at a spacing of 1 µm. Recordings at the permissive temperature of 15°C comprised 1500 scans owing to the slower pace of development. All parameters including scans using fluorescent channels were programmed for specific time points using the imaging software Caenotec. Manual lineaging was performed as described by Schnabel et al. (1997) using the Simi BioCell software (Simi Reality Motion, Unterschleissheim, Germany) software. The microenvironment under the objective was kept at a constant temperature via either an F12-ED or CD-200F Refrigerated/Heating Circulator (Julabo, Seelbach, Germany) and a bespoke copper collar surrounding the 100× objective.

### Temperature-shift experiments

Temperature-shift experiments were conducted using the same setup as for temperature controlled 4D-lineaging described in the ‘4D-lineaging’ section above. Temperature downshift recordings were started at the non-permissive temperature 25°C and the temperature of the heated water circulator was changed to 15°C for the downshift after a set duration. The opposite was true for upshifts, which began at the permissive temperature 15°C and were then changed to the non-permissive 25°C. Data points were fitted to a Boltzmann sigmoid curve. Here, ‘Bottom’ refers to the lowest value of the curve, ‘Top’ to the highest and ‘V50’ the middle of the curve:
(1)

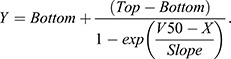
The temperature-sensitive period (Suzuki, 1970) or *tcrit* (Hirsh and Vanderslice, 1976) was defined as starting at the point on the downshift at which the percentage mutant phenotype is first non-zero. The end was defined as the point on the upshift at which the percentage mutant phenotype first reaches zero.

### 4D-lineage-based screen of embryonic-lethal, temperature-sensitive mutants

A forward genetic screen employing EMS mutagenesis was performed in accordance with standard protocols (Brenner, 1974). Mutants that were temperature sensitive at 25°C and did not fail at the very earliest stages of embryogenesis constituted the secondary screening population. These embryos were manually 4D-lineaged as described in the ‘4D-lineaging’ section above. Mutants of interest were identified as those that phenocopied the precocious Caapa division of *hlh-14* mutants, or otherwise displayed C lineage aberrations.

### Cell volume and area measurements

Cell pseudo-volumes were calculated from measured cell areas in DIC *z*-stack images using the image processing software Fiji (Schindelin et al., 2012). Stacks were acquired from the 4D-lineaging setup. Areas were measured by tracing the extent of the cell in an image. The volume between slices of the *z*-stack was calculated as a truncated cone with the slices known to be spaced 1 µm apart. The radii were calculated from circles of equivalent volume to those of measured cell areas. The volumes between slices were summed to approximate cell volume and volume ratios between daughter cells were always expressed with respect to that of the posterior daughter; for example, 1.5 (1.5:1 A:P) or 0.85 (0.85:1 A:P, equivalent to 1:1.18). The volume of a truncated cone is therefore given as:
(2)

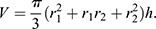
Cell nuclei areas were measured in Fiji using maximum intensity projections of GFP images of the *gmIs20[hlh-14prom::hlh-14::gfp]* transgene.

### Statistical analysis and genotypes

Statistical analyses were preformed using GraphPad Prism or Microsoft Excel. Absolute cell volumes, cell volume ratios, division timing and cell cycle ratios were compared using one-way ANOVA analysis in GraphPad Prism, as there were more than three groups compared and all group comparisons were computed. As group sample sizes were fewer than 50, Tukey's HSD test was used in post-hoc testing and correction for multiple comparisons. When only two groups were compared, unpaired two-tailed *t*-tests were conducted in Microsoft Excel. F-tests were carried out first to check for equal or unequal variances.

The *pig-1*, *ham-1* and *let-19* mutant genetic backgrounds of samples analysed for cell volumes and cell volume ratios included either *gmIs20 [hlh-14prom::hlh-14::gfp rol-6(+)] II* alone or in combination with *otIs458 [ceh-63prom::gfp] III; stIs10166 [dpy-7p::HIS-24::mCherry,unc-119(+)]*. Therefore, the ‘wild-type’ samples in such cases comprised a combination of these same transgene backgrounds. There was no significant difference between these backgrounds for the cell volume ratios analysed (Fig. S11). As the *hlh-14(tm295)* mutant did not carry the transgenes, N2 was used as the wild-type control strain for *hlh-14(tm295)*.

When phenotypes were assessed in the same embryos, the disparity between sample sizes for each phenotype is explained as follows. Division timing data for all mutant and wild-type samples was collected in backgrounds containing *gmIs20 [hlh-14prom::hlh-14::gfp rol-6(+)]* [with the exception of *hlh-14(tm295)* and N2]. A subset of these embryos had appropriately timed imaging to assess *hlh-14* expression in Caapa (the DVC neuroblast); a further subset again had appropriate image quality to allow cell volume measurements. As such, there is a decreasing sample size for these phenotypes from the same set of embryos.

### Mapping and whole-genome sequencing

Genomic DNA preparation was performed using the Gentra Puregene Tissue Kit (QIAGEN) in accordance with the manufacturer's supplemental nematode protocol. Library preparation was performed by the UCL Genomics team at the UCL Great Ormond Street Institute of Child Health, London, UK. Mapping of causal mutations was performed via the ‘Hawaiian Cross’ mapping-by-sequencing method. Strains were crossed to the highly polymorphic Hawaiian CB4856 strain and linkage of N2 regions was used to locate the causal mutant locus (Doitsidou et al., 2016). Mutant genome analysis was performed using the Galaxy web server-based (Afgan et al., 2018) pipeline CloudMap (Minevich et al., 2012). The Galaxy server was maintained by R.J.P.

### Complementation and rescue of *let-19 a*lleles

Complementation crosses were performed to confirm non-complementation of the *t3200* and *t3273* alleles. Further crosses confirmed non-complementation between *t3200* and another known *let-19* allele, *t3219.* Non-complementation between *t3219* and the mnDf46 deficiency, which covers only the *let-19* and *rol-6* genes, had confirmed *t3219* as a *let-19* allele. Either the GFP marker *ceh-63::gf* or *flp-10::gfp* was first crossed into the males so that cross progeny could be identified and only those scored for lethality. Crosses were performed at 15°C in both directions so that males of each mutant were crossed to the hermaphrodites of the opposite genotype. Gravid hermaphrodites from complementation crosses were singled and incubated at 25°C so that F1 embryos developed at the non-permissive temperature. To investigate maternal effect, crosses were performed between wild types and each of the mutants. Rescue of embryonic lethality for *t3200* and *t3273* was performed using the WRM061cD04 fosmid, covering the region including the *let-19* locus and six further genes, three pseudo genes and a non-coding RNA. The sequence for WRM061cD04 is publicly available from the nematode research community database (https://wormbase.org/species/c_elegans/sequence/WRM061cD04#04--10). Mutant strains containing the fosmid as an extra-chromosomal array fused to *myo-2::gfp* were built. P0s were singled to 25°C and lethality was scored for F1 embryos with or without the array; presence was indicated by GFP.

### Image processing

Imaging data, including *z*-stack construction and maximum intensity projections, were processed using Fiji, a distribution of ImageJ (Schindelin et al., 2012).

## Supplementary Material



10.1242/develop.200981_sup1 Supplementary information

## The people behind the papers

This article has an associated ‘The people behind the papers’ interview with some of the authors.
