# Supplementary material for: Control of successive unequal cell divisions by neural cell fate regulators determines embryonic neuroblast cell size
Source: Development. 2024 Feb 5;151(3):dev200981. doi: 10.1242/dev.200981 (PMC10911278; doi:10.1242/dev.200981)
Supplement: Supplementary information [file develop-151-200981-s1.pdf]

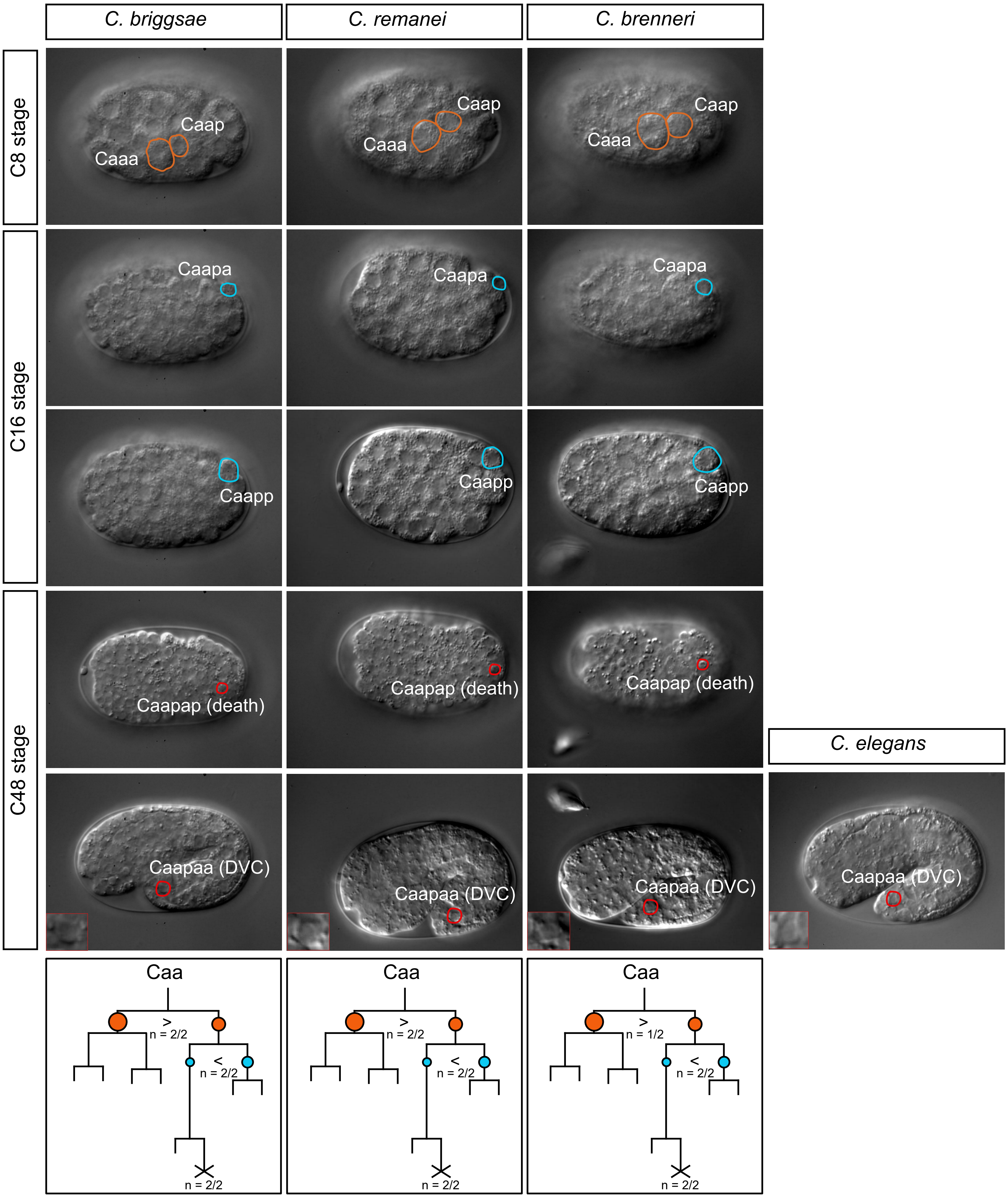

Development • Supplementary information

Fig. S1. Unequal cleavages in the C lineage in other *Caenorhabditis* species DIC images of the Caa and Caa cleavages, and the Caapap cell death in *C. briggsae, C. remanei* and *C. brenneri*. The Caa cleavage daughters are indicated in orange, the Caap daughter in blue, and Caapa daughters in red. Two images presented when the cells are on different imaging planes. For Caapaa (DVC) an enlarged image is included inset. Cell lineage diagrams of the neurogenic branch based on manually 4D-lineaged embryos, branch length represents division timing. Circles indicate relative size of cells; < or > representing the bias in the unequal cleavage. X represents a cell death. n numbers indicate the number of lineaged embryos displaying the indicated unequal cleavage.

Development • Supplementary information

Figure S2

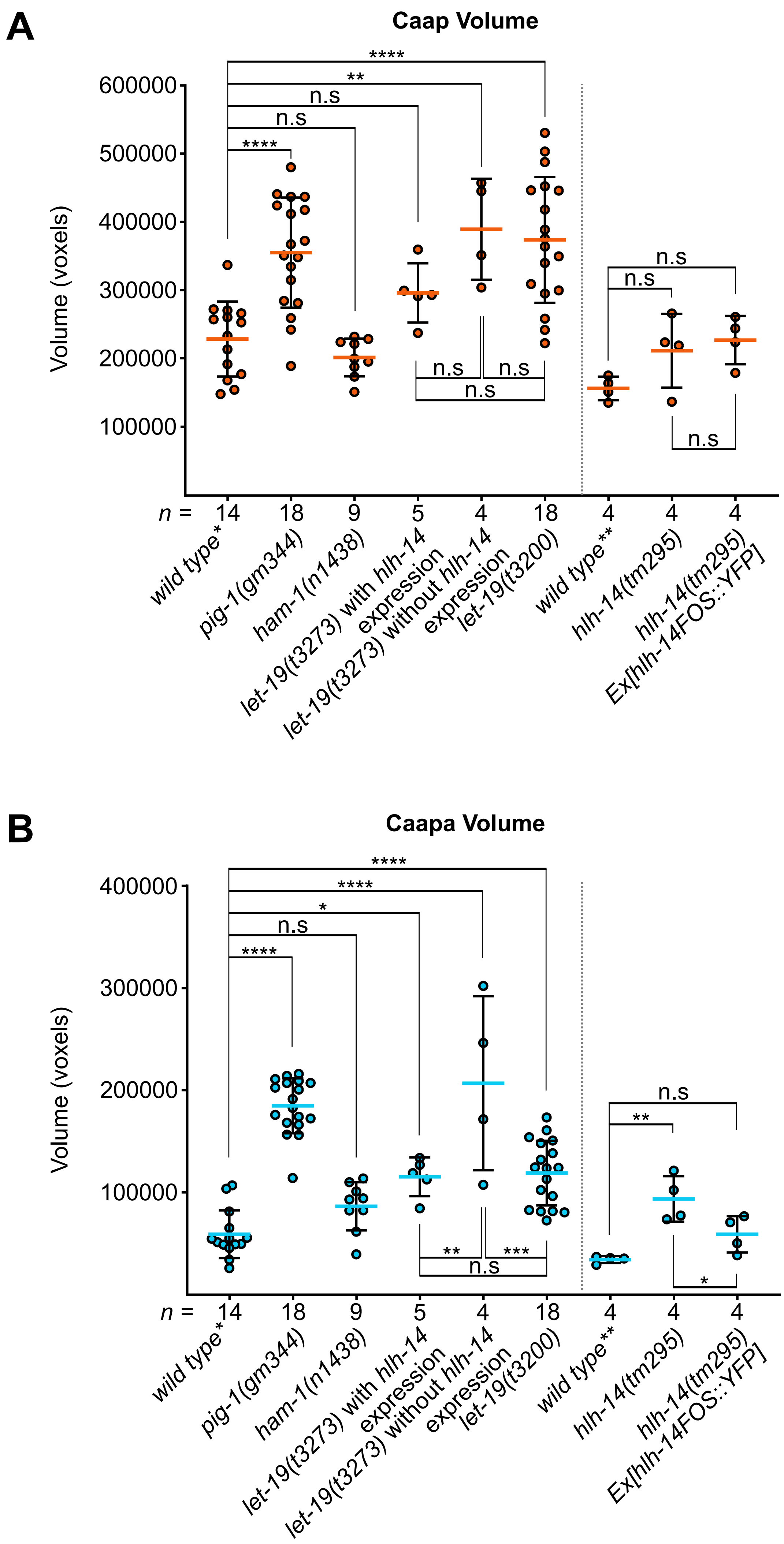
A
Caap Volume
Volume (voxels)
600000
500000
400000
300000
200000
100000
****
**
n.s
n.s
****
n.s
n.s
n.s
n.s
n.s
n.s
n =
14
18
9
5
4
18
4
4
4
wild type*
pig-1(gm344)
ham-1(n1438)
let-19(t3273) with hlh-14 expression
let-19(t3273) without hlh-14 expression
let-19(t3200)
wild type**
hlh-14(tm295)
hlh-14(tm295) Ex[hlh-14FOS::YFP]
B
Caapa Volume
Volume (voxels)
400000
300000
200000
100000
****
****
*
n.s
****
**
***
n.s
n.s
**
*
n =
14
18
9
5
4
18
4
4
4
wild type*
pig-1(gm344)
ham-1(n1438)
let-19(t3273) with hlh-14 expression
let-19(t3273) without hlh-14 expression
let-19(t3200)
wild type**
hlh-14(tm295)
hlh-14(tm295) Ex[hlh-14FOS::YFP]

Fig. S2. Caap and Caapa absolute volumes

Dot plots of (A) Caap and (B) Caapa absolute volumes (the smaller daughters in *wild type* cleavages) in all strains measured in the study, in voxels, with means and S.D. For *let- 19(t3273)* the proportion of embryos that express *hlh-14* are plotted separately from those that do not. Grey dotted line indicates the separate groups of genotypes compared (those with and without transgenes) and their appropriate *wild type* genotypes. Caap in orange, Caapa in blue as in all other figures. For clarity only comparisons to *wild type* controls and within each mutant group are illustrated (all comparisons computed). n.s not significant, * $p<0.05$, ** $p<0.01$, *** $p<0.001$, **** $p<0.0001$ (one-way ANOVA with Tukey's HSD). *wild type* = hlh-14 [gmIs20] II* and *hlh-14 [gmIs20] II; dpy-7 [stIs10166]; ceh-63 [otIs458] III*, *wild type*** = N2. For further details on *wild type* and mutant genotypes, refer to the materials and methods section.

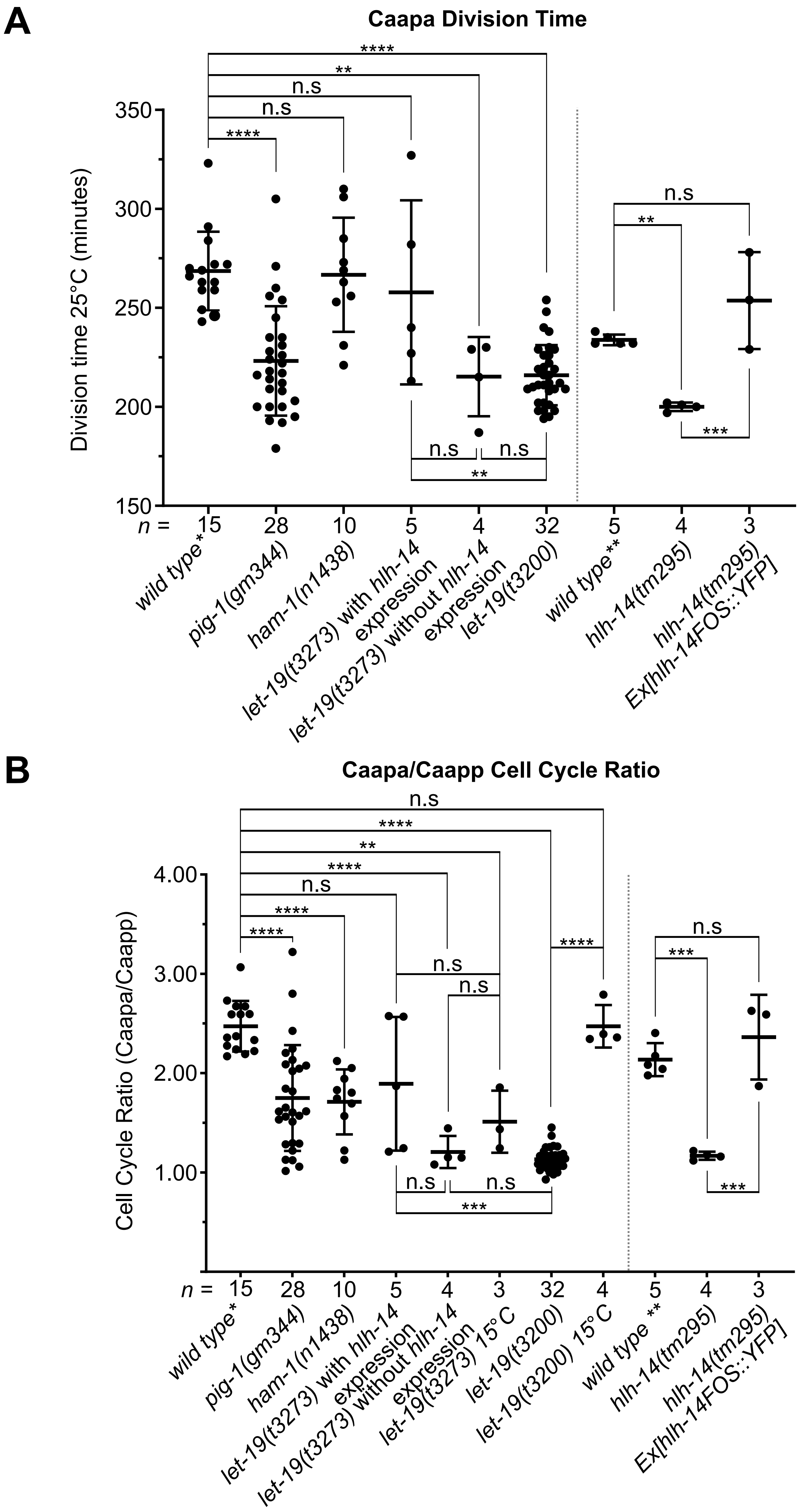

Development • Supplementary information

Fig. S3. Caapa division times and Caapa/Caapp cell cycle ratios

Dot plots of (A) Caapa division time in minutes at 25°C and (B) Caapa/Caapp cell cycle duration ratio (CCR) in all strains measured for cell volume in the study, with means and S.D. For *let-19(t3273)* the proportion of embryos that express *hlh-14* are plotted separately from those that do not. Grey dotted line indicates the separate groups of genotypes compared (those with and without transgenes) and their appropriate *wild type* genotypes. Caap in orange, Caapa in blue as in all other figures. For clarity only comparisons to *wild type* controls and within each mutant group are illustrated (all comparisons computed). n.s not significant, ** $p<0.01$, *** $p<0.001$, **** $p<0.0001$ (one-way ANOVA with Tukey's HSD). *wild type** = *hlh-14 [gmIs20] II* and *hlh-14 [gmIs20] II; dpy-7 [stIs10166]; ceh-63 [otIs458] III*, *wild type*** = N2. For further details on *wild* type and mutant genotypes, refer to the materials and methods section

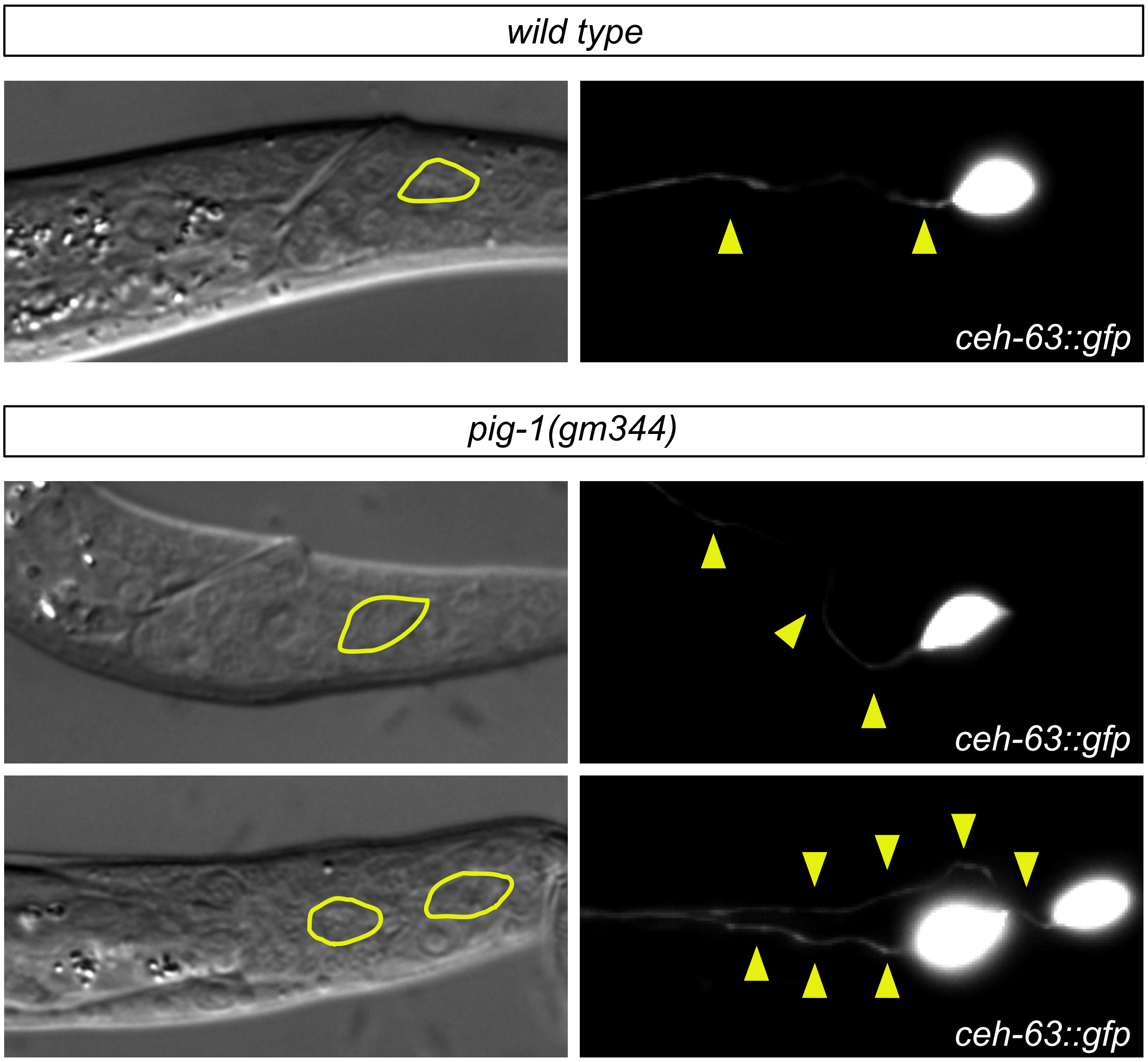

Fig. S4. DVC neuron morphology in *pig-1(gm344)* L1 larvae

DIC and GFP images of *ceh-63 [otIs458]* expression in DVC neurons, and so neuronal morphology in hatched *wild type* and *pig-1(gm344)* larvae at the L1 stage. Yellow lines = outline of cell in DIC, yellow arrows = neuronal processes.

Development • Supplementary information

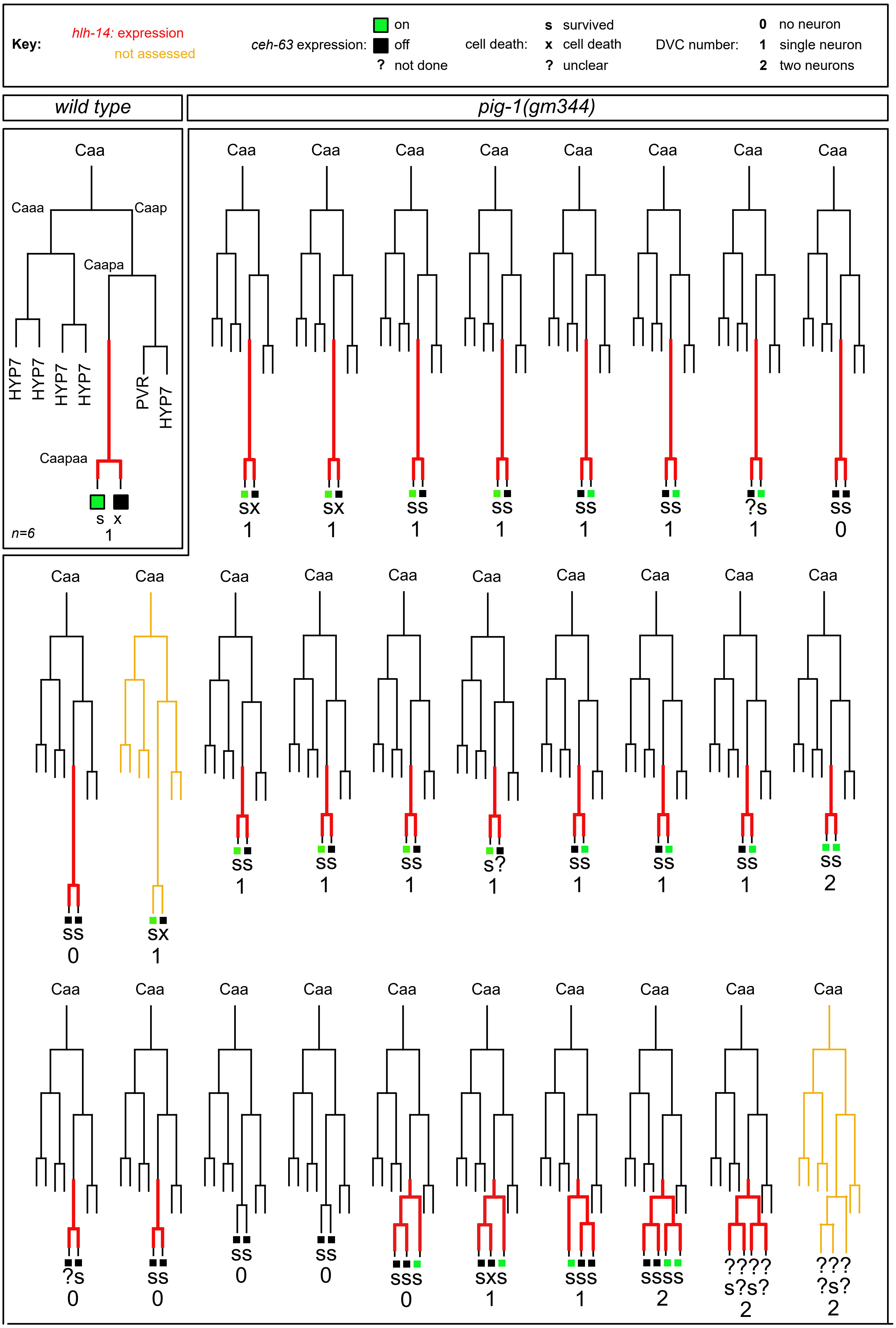

Development • Supplementary information

Fig. S5. Individual lineages of *pig-1(gm344)* mutants

Lineage diagrams of the neurogenic branch of the C lineage in *wild type* and individual lineaged *pig-1(gm344)* embryos. Branch lengths indicate division times such that precocious divisions are indicated. Red lines represent *hlh-14::gfp [gmIs20]* expression, orange represents embryos in which *hlh-14* expression was not assessed. Terminal phenotypes are represent below each lineage: squares represent DVC neuroblast daughters; green = the expression of *ceh-63 [otIs458]* and DVC fate, black = no expression, ? = unscored. X = cell death , s = survived, ? = unclear/cell lost during lineaging. Number = DVC number as indicated by *ceh-63 [otIs458]* expression.

Development • Supplementary information

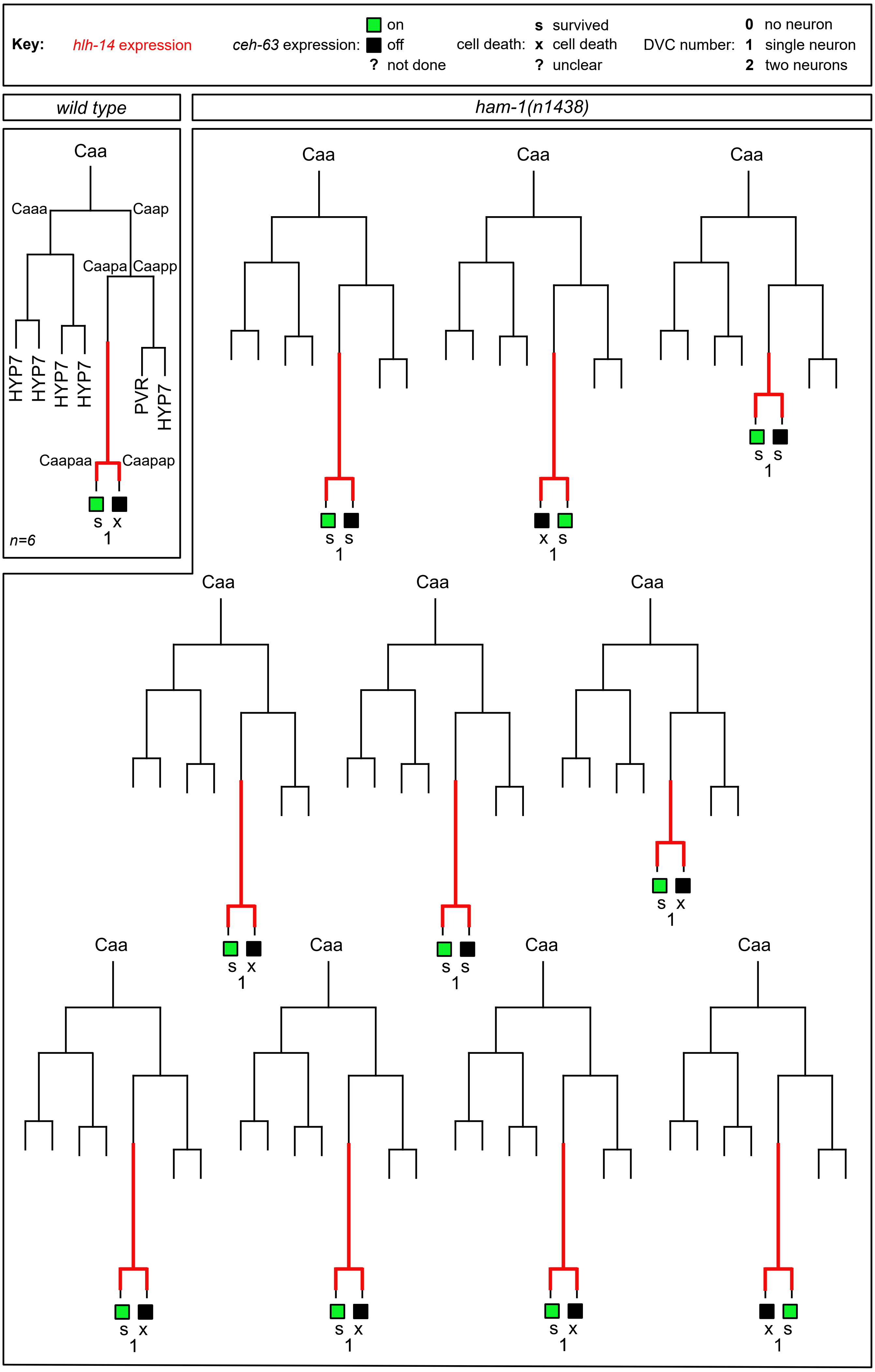

Development • Supplementary information

Fig. S6. Individual lineages of *ham-1(n1438)* mutants

Lineage diagrams of the neurogenic branch of the C lineage in *wild type* and individual *ham-1(n1438)* embryos. Branch lengths indicate division times such that precocious divisions are indicated. Red lines represent *hlh-14::gfp [gmIs20]* expression, orange represents embryos in which *hlh-14* expression was not assessed. Terminal phenotypes are represent below each lineage: squares represent DVC neuroblast daughters; green = the expression of *ceh-63 [otIs458]* and DVC fate, black = no expression, ? = unscored. X = cell death , s = survived, ? = unclear/cell lost during lineaging. Number = DVC number as indicated by *ceh-63 [otIs458]* expression.

Development • Supplementary information

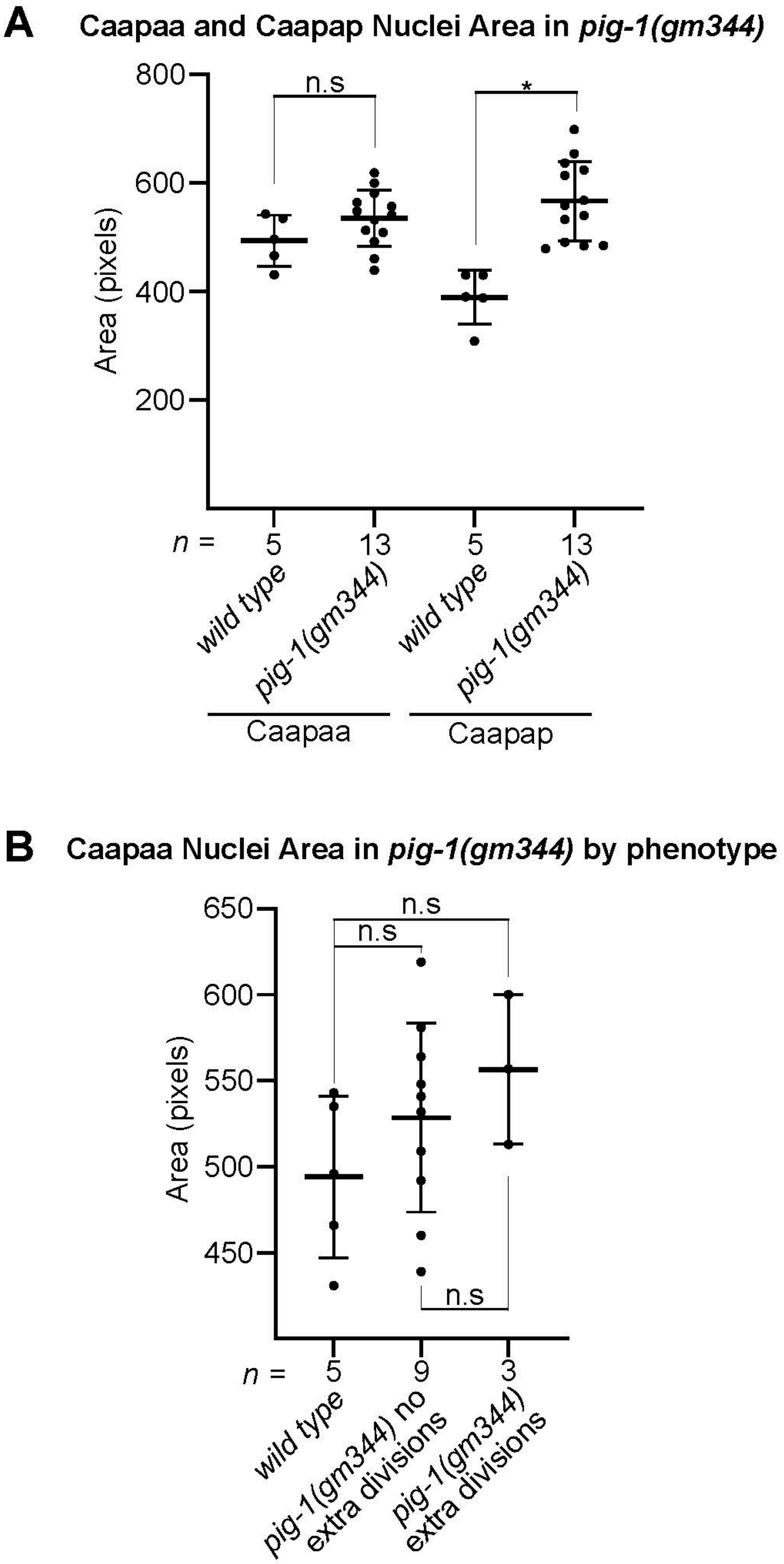

Development • Supplementary information

Fig. S7. Caapaa and Caapap nuclei areas in *pig-1(gm344)* mutants

(A) Dot plots of Caapaa and Caapap nuclei areas in pixels in *wild type [gmIs20]* and *pig- 1(gm344)* embryos.

n.s = not significant, * = p<0.05 (unpaired, two-tailed t-*t*est). (B) Dot plot of Caapaa nuclei areas in *wild type* embryos and *pig-1(gm344)* embryos plotted separately for whether Caapaa divided again or did not.

n.s = not significant (one-way ANOVA with Tukey's HSD). For details on *wild type* and mutant genotypes, refer to the materials and methods section.

Development • Supplementary information

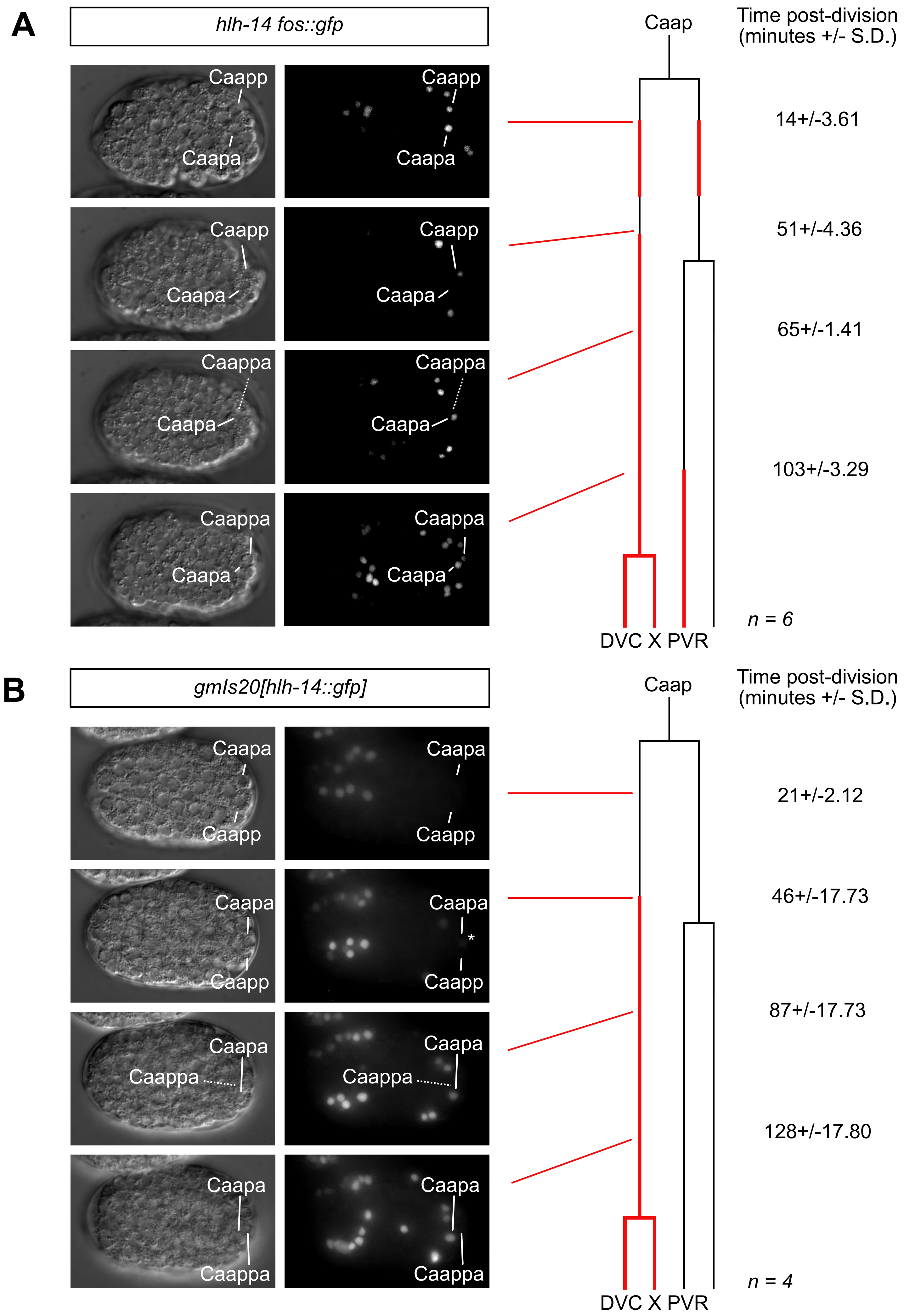
A
hlh-14 fos::gfp
Caapp
Caapa
Caappa
Caap
Time post-division
(minutes +/- S.D.)
14+/-3.61
51+/-4.36
65+/-1.41
103+/-3.29
n = 6
DVC X PVR
B
gmIs20[hlh-14::gfp]
Caapa
Caapp
Caappa
*
Caap
Time post-division
(minutes +/- S.D.)
21+/-2.12
46+/-17.73
87+/-17.73
128+/-17.80
n = 4
DVC X PVR

Fig. S8. Expression of *hlh-14* transgenes following Caap division

DIC and GFP maximum intensity images from single embryos of *hlh-14* expression, together with lineage diagram for (A) *hlh-14 [gmIs20]* and (B) *hlh-14 fosmid::gfp* transgenes. Cell names are indicated with labels and lines, dotted lines indicate cell position on another plane in DIC. On lineage diagrams branch lengths are indicative to cell cycle duration, red indicated *hlh-14* expression, beginning at the earliest detected time point. Expression patterns are constructed from a consensus of all lineage embryos at 25°C. The fading of *hlh-14 fosmid::gfp* expression at the second timepoint is evident with expression in Caapp fading and Caapa not detected. Timings for the four timepoints shown are indicated as minutes post-division of Caap ± standard deviation.

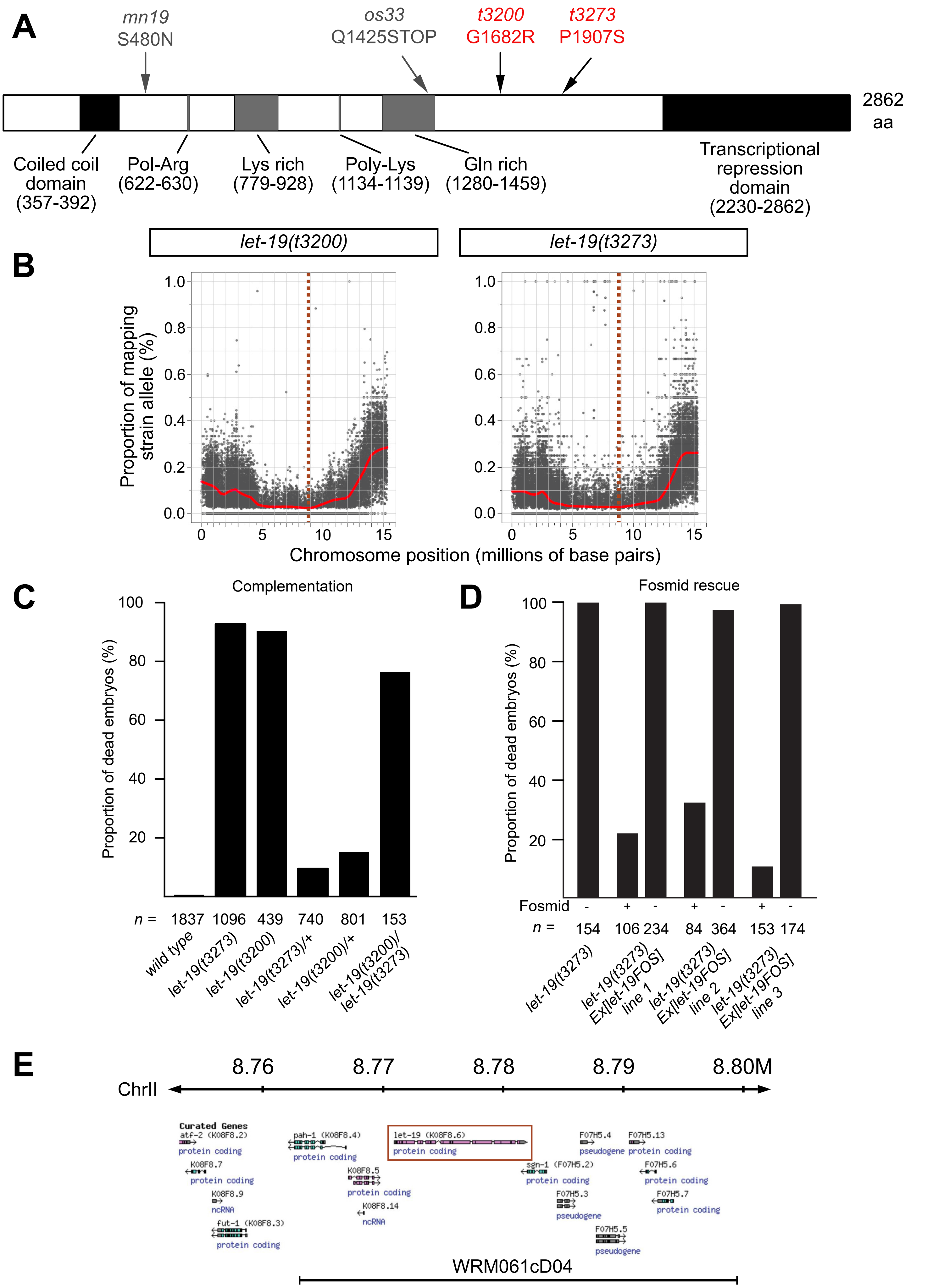
A
mn19
S480N
os33
Q1425STOP
t3200
G1682R
t3273
P1907S
2862 aa
Coiled coil domain (357-392)
Pol-Arg (622-630)
Lys rich (779-928)
Poly-Lys (1134-1139)
Gln rich (1280-1459)
Transcriptional repression domain (2230-2862)
B
let-19(t3200)
let-19(t3273)
Proportion of mapping strain allele (%)
Chromosome position (millions of base pairs)
C
Complementation
Proportion of dead embryos (%)
n = 1837 1096 439 740 801 153
wild type
let-19(t3273)
let-19(t3200)
let-19(t3273)/+
let-19(t3200)/+
let-19(t3200)/ let-19(t3273)
D
Fosmid rescue
Proportion of dead embryos (%)
Fosmid - + - + - + -
n = 154 106 234 84 364 153 174
let-19(t3273)
let-19(t3273) Ex[let-19FOS] line 1
let-19(t3273) Ex[let-19FOS] line 2
let-19(t3273) Ex[let-19FOS] line 3
E
ChrII
8.76
8.77
8.78
8.79
8.80M
Curated Genes
let-19 (K08F8.6)
WRM061cD04

Fig. S9. Mapping, complementation and rescue of *t3200* and *t3273* as alleles of *let-19*

(A) Diagram of the *let-19* protein structure. Black shading indicates identified domains; grey shading indicates an area rich in the indicated amino acid. The alleles identified in this study and their resultant amino acid changes are indicated in red; other known alleles are indicated in grey. (B) Mapping graphs for *t3200* and *t3273*. The Y-axis is the proportion of the sample containing SNPs from the mapping strain (Hawaiian), a measure of heterozygosity. A value of 1.0 would be entirely Hawaiian, 0.0 entirely from the mutant strain. A red dotted line indicates the location of the causal lesion in the *let-19* locus. (C) A bar chart of the quantification of F1 embryonic or larval lethality at the non-permissive temperature of 25°C from complementation tests between the *t3200* and *t3273* alleles. The graph includes quantification of lethality in maternal effect tests from *wild types* crossed into the mutants. (D) A bar chart of the quantification of F1 embryonic lethality following fosmid rescue. Embryonic lethality scored in three lines containing the WRM061cD04 fosmid covering the *let-19* locus; + indicates the presence of the fosmid, - indicates its absence. For both *t3200* and *t3273* strains, *let-19* was the only gene containing a lesion covered by WRM061cD04. (E) Diagram of the region on chromosome II containing the *let-19* locus, adapted from the genome browser accessed via wormbase.org. The *let-19* locus is indicated by a red box. The region covered by the WRM061cD04 fosmid is indicated below the depiction of the genes in the region.

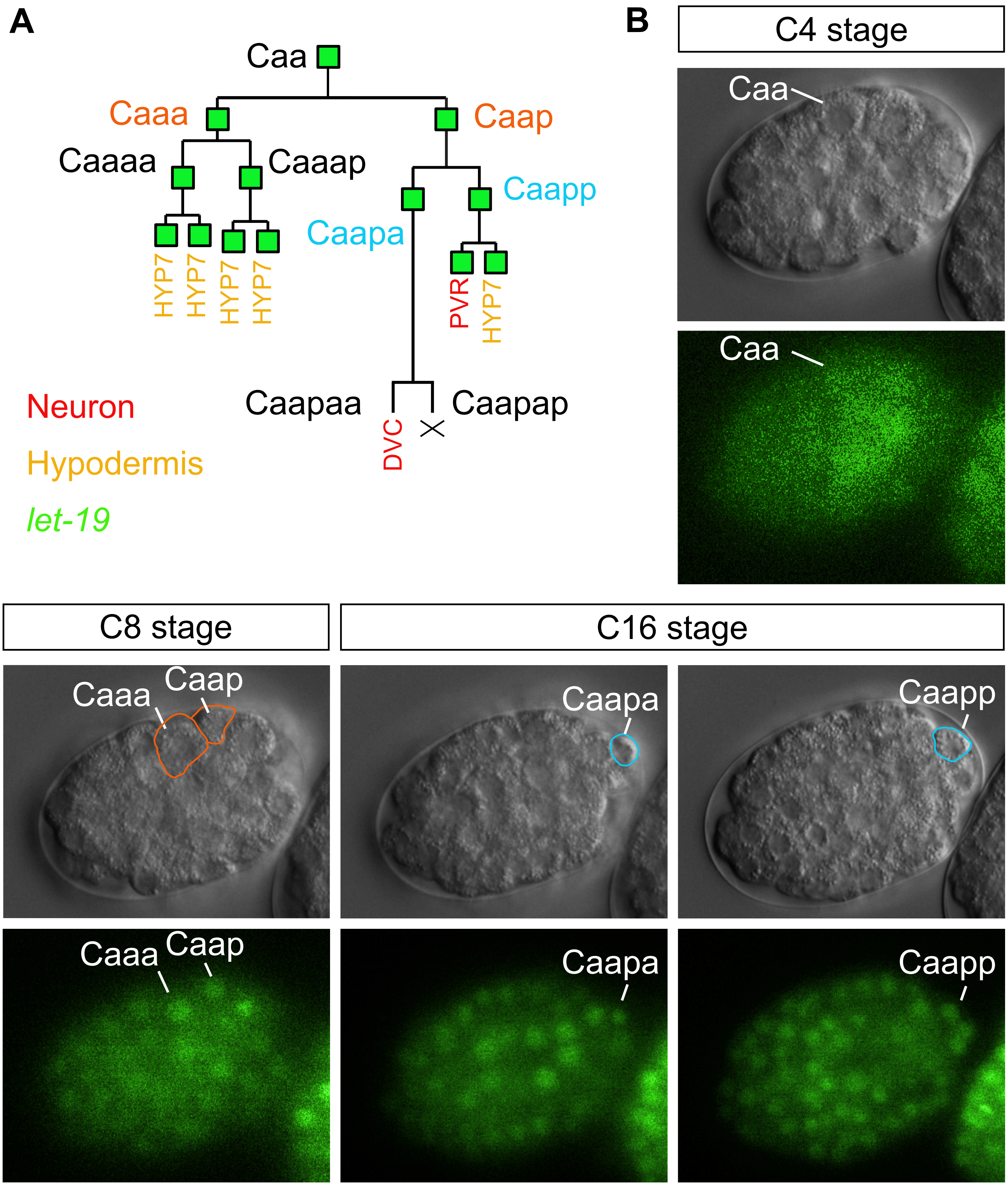
A
Caa
Caaa
Caap
Caaaa
Caaap
Caapa
Caapp
HYP7
HYP7
HYP7
HYP7
PVR
HYP7
Caapaa
Caapap
DVC
Neuron
Hypodermis
let-19
B
C4 stage
Caa
Caa
C8 stage
C16 stage
Caaa
Caap
Caapa
Caapp
Caaa
Caap
Caapa
Caapp

Fig. S10. Expression of *let-19* in the C lineage cleavages

(A) Lineage diagram of the C lineage branch descended from Caa. Squares indicate cells scored for expression of *let-19*, solid green indicating expression. Red text indicates neurons, yellow indicates hypodermis. The cell names of Caa daughters are in orange, Caap daughters in blue, as in other figures. (B) DIC and GFP images of *let-19* expression in the mother and daughter cells of the unequal cleavages studied (Caa, Caaa. Caap. Caapa, Caapp). Caa daughters are indicated in orange, Caap daughters in blue, as in all other figures.

Development • Supplementary information

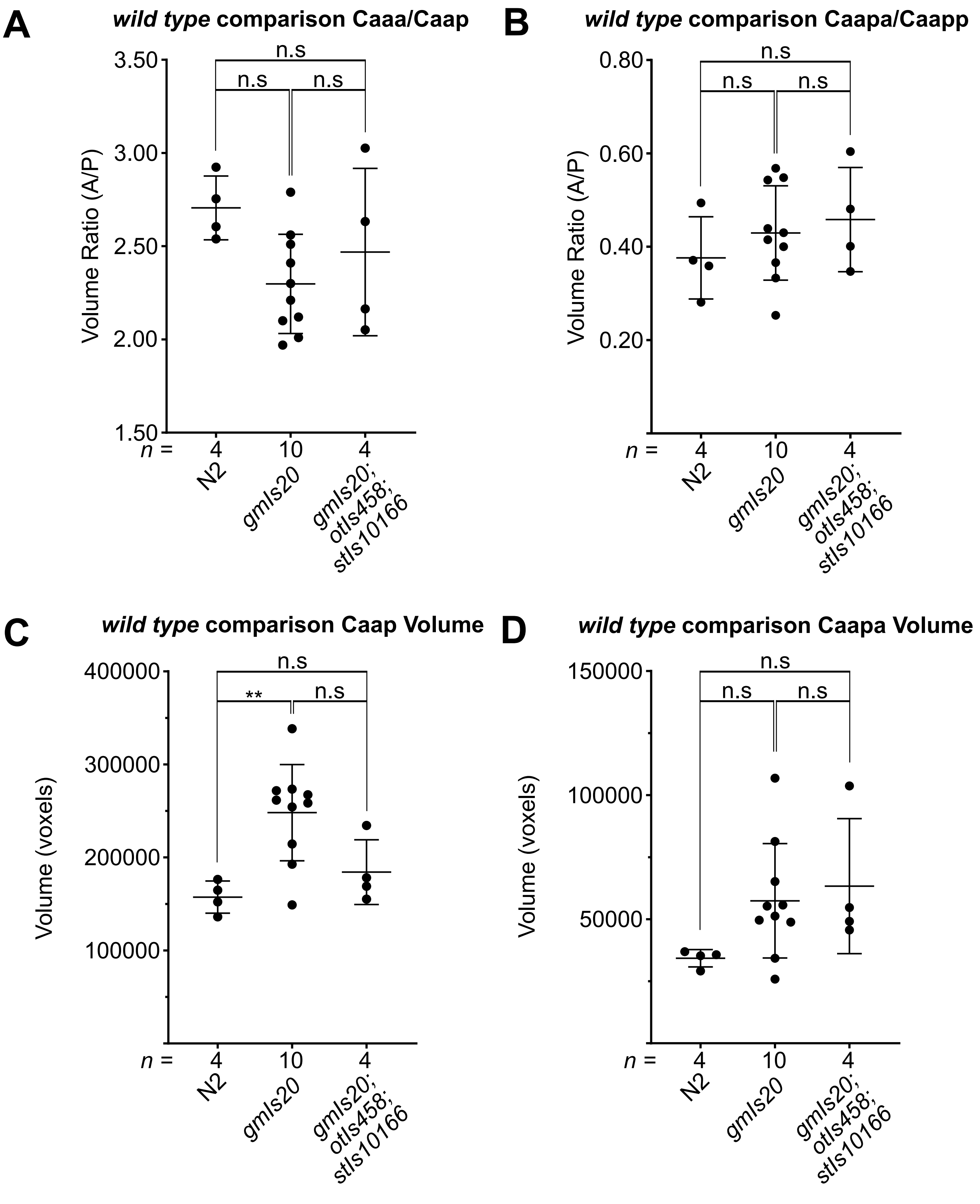

Fig. S11. Comparison of Caa and Caap division ratios and Caap and Caapa absolute volumes in *wild type* genotypes
Dot plots of the volumetric ratio of the (A) Caa and (B) Caap cleavages in N2, *hlh-14 [gmIs20]* carrying and *hlh-14 [gmIs20], dpy-7 [stIs10166], ceh-63 [otIs458]* transgene carrying embryos expressed as the anterior daughter volume/posterior daughter volume, with means and S.D. n.s = not significant (one-way ANOVA with Tukey's HSD). Dot plots of absolute volume of (C) Caap and (D) Caapa in voxels in N2, *hlh-14 [gmIs20]* carrying and *hlh-14 [gmIs20], dpy-7 [stIs10166], ceh-63 [otIs458]* transgene carrying embryos. n.s = not significant, ** = p<0.01 (one-way ANOVA with Tukey's HSD).

Table S1. Strains used

**Strain number**	**Genotype**
C. elegans	
CB4856	*Hawaiian*
CHL5	*otIs458[ceh-63prom::gfp] III; otIs92[flp-10prom::gfp]*
CHL28	*stIs10166 [dpy-7p::HIS-24::mCherry + unc-119(+)]; let-19(t3200) gmIs20 [hlh-14prom::hlh-14::gfp rol-6(+)] II; otIs458 [ceh-63::gfp] III*
CHL22	*let-19(t3200) gmIs20 [hlh-14prom::hlh-14::gfp rol-6(+)] II*
CHL31	*stIs10166 [dpy-7p::HIS-24::mCherry + unc-119(+)]; gmIs20 [hlh-14prom::hlh-14::gfp rol-6(+)] II; otIs458 [ceh-63::gfp] III*
CHL40	*let-19(t3273); drpEx1[WRM061Cd04, myo-2 prom::gfp rd 2.3.2 (line1)*
CHL41	*let-19(t3273); drpEx2[WRM061Cd04, myo-2 prom::gfp rd 4.3.1 (line2)*
CHL42	*let-19(t3273); drpEx3[WRM061cD04, myo-2 prom::gfp rd 3.1 (line3)*
CHL51	*gmIs20 [hlh-14prom::hlh-14::gfp rol-6(+)] II; otIs458 [ceh-63::gfp] III; pig-1(gm344) IV*
CHL52	*stIs10166 [dpy-7p::HIS-24::mCherry + unc-119(+)]; gmIs20 [hlh-14prom::hlh-14::gfp rol-6(+)] II; otIs458 [ceh-63::gfp] III, pig-1(gm344) IV*
CHL53	*stIs10166 [dpy-7p::HIS-24::mCherry + unc-119(+)]; gmIs20 [hlh-14prom::hlh-14::gfp rol-6(+)] II; otIs458 [ceh-63::gfp] III; ham-1(n1438) IV*
CHL54	*let-19(t3273) II gmIs20 [hlh-14prom::hlh-14::gfp rol-6(+)] II*
CHL55	*let-19(t3273) II; otIs458 [ceh-63::gfp] III*

Development • Supplementary information

GE4421	*let-19(t3200) II*
GE4547	*let-19(t3219) II*
HS616	*osEx108 [(pAY105) let-19::GFP + rol-6(su1006)]*
GE4634	*let-19(t3273) II*
MD3998	*bcSi43[pig-1::gfp] II; unc-119(ed3) III*
MT3351	*ham-1(n1438) IV*
N2	*wild type*
NG4080	*gmIs20 [hlh-14prom::hlh-14::gfp rol-6(+)] II*
NG4280	*hlh-14(tm295)/mln1 II [mIs14 dpy-10(e128)]*
OH10173	*Ex[hlh-14FOS(WRM0627dH07)::yfp; rol-6(d)]*
OH11974	*otIs458 [ceh-63::gfp] III*
SD1546	*stIs10166 [dpy-7p::HIS-24::mCherry + unc-119(+)]*
SP582	*unc-4(e120) mnDf46/mnC1 dpy-10(e128) unc-52 (e444)II*
MD3876	*pig-1(gm344) IV*
MD3877	*pig-1(tm1510) IV*
CHL17	*stIs10166 [dpy-7p::HIS-24::mCherry + unc-119(+)]; let-19(t3273) II*
OH15657	*otIs713[hlh-14 fosmid::gfp; ttx-3 prom::rfp]*
CHL154	*pig-1(gm344) IV; otIs713[hlh-14 fosmid::gfp; ttx-3 prom::rfp]*
CHL155	*pig-1(tm1510) IV; otIs713[hlh-14 fosmid::gfp; ttx-3 prom::rfp]*
**Other *Caenorhabditis* species**	
AF16	*C. briggsae*
CB5161	*C. brenneri*
EM646	*C. remanei ssp. vulgaris*
