# Supplementary material for: Control of successive unequal cell divisions by neural cell fate regulators determines embryonic neuroblast cell size
Source: Development. 2024 Feb 5;151(3):dev200981. doi: 10.1242/dev.200981 (PMC10911278; doi:10.1242/dev.200981)
Supplement: Supplementary file 1 [file develop-151-200981_review_history.pdf]

Control of successive unequal cell divisions by neural cell fate regulators determines embryonic neuroblast cell size

Thomas W. Mullan, Terry Felton, Janis Tam, Osama Kasem, Tim J. Yeung, Nadin Memar, Ralf Schnabel and Richard J. Poole
DOI: 10.1242/dev.200981

Editor: Swathi Arur

Review timeline

Original submission:	25 May 2022
Editorial decision:	28 June 2022
First revision received:	13 December 2023
Accepted:	2 January 2024

Original submission

First decision letter

MS ID#: DEVELOP/2022/200981

MS TITLE: Progressive control of unequal cell divisions by neural cell fate regulators determines embryonic neuroblast cell size

AUTHORS: Thomas W Mullan, Terry Felton, Janis Tam, Osama Kasem, Justina Yeung, Nadin Memar, Ralf Schnabel, and Richard J Poole

I have now received all the referees' reports on the above manuscript, and have reached a decision. As you will see, the referees express considerable interest in your work, and also provide recommendations both textual and experimental to greatly clarify the manuscript. If you are able to revise the manuscript along the lines suggested, which may involve further experimentation, I will be happy receive a revised version of the manuscript. Your revised paper will be re-reviewed by one or more of the original referees. If it would be helpful, you are welcome to contact us to discuss your revision in greater detail. Please send us a point-by-point response indicating your plans for addressing the referee's comments, and we will look over this and provide further guidance.

Please attend to all of the reviewers' comments and ensure that you clearly highlight all changes made in the revised manuscript. Please avoid using 'Tracked changes' in Word files as these are lost in PDF conversion. I should be grateful if you would also provide a point-by-point response detailing how you have dealt with the points raised by the reviewers in the 'Response to Reviewers' box. If you do not agree with any of their criticisms or suggestions please explain clearly why this is so.

Reviewer 1

Advance summary and potential significance to field

In this paper, the authors describe for the first time that two unequal cell divisions precede the birth of the DVC neuron in the C lineage of C. elegans embryo, a fate that distinguishes this cell from its lineage-related hypodermal cells. The authors set out to answer the question of whether these cell size variations in the DVC progenitors are driving the acquisition of the neural fate in this branch of the lineage. By preventing asymmetric cell division, they convincingly demonstrate that

© 2024. Published by The Company of Biologists under the terms of the Creative Commons Attribution License (https://creativecommons.org/licenses/by/4.0/).

the variation in cell size in the DVC progenitors does not cause cell fate defects, although it can lead to abnormalities in the number and anterior-posterior specification of the neuronal progeny. Finally, the authors discover that two different proneural genes expressed sequentially in the DVC progenitors have an effect on controlling progenitor cell size, besides being required for DVC specification. The authors propose the concomitant regulation of neural fate and cell size as a general mechanism to ensure adequate neuronal size during C. elegans embryonic development, where cell growth mechanisms do not operate.

The article is logically structured and easy to follow, and its main conclusions are supported by convincing, well-presented data. However, a series of comments should be clarified in order to improve the manuscript.

Comments for the author

- The authors demonstrate not only that DVC progenitors suffer repeated asymmetric cell divisions to generate a smaller-sized neuron, but also show that this size bias is evolutionarily conserved across the C lineage of other related nematodes. However, it is not completely clear if the neural fate of the Caapa progeny is also conserved in these related species, or whether onnly the asymmetries are conserved.

- The authors discuss that the proneural genes hlh-14 and let-19 act concomitantly to control the formation of a DVC neuron of the adequate size, and argue for the requirement of cellular mechanisms that control cell size at the level of cell division in an embryo where cell growth/shrinkage is not permitted.
However, although the effect of abnormal cell size in cell cycle and neuronal progeny number is shown, the possible effects of a larger neuroblast in function/morphology of the resulting DVC neuron are not experimentally tackled or discussed.
o DVC neuron size (volume) is not measured. If technically possible, this measurement should be quantified for hlh-14 and let-19 mutants.
o An assessment of adult morphology and function of DVC in mutant animals if technically possible, would provide a better picture of the relevance of the neuroblast size control processes described in this article.

- The Caap neuroblast produces two neurons, DVC from the Caapa neuroblast and PVR from the Caapp neuroblast, and in both cases the proneural gene hlh-14 is activated. However, how the different mutant conditions (pig-1, ham-1, hlh-14 and let-19) affect the fate of PVR neuron is not analyzed or discussed. The analysis of PVR fate would help generalize the conclusions of the study regarding the effect of cell size on neuronal fate and the let-19 requirement for downstream hlh-14 expression.
Understanding that a study of PVR fate with specific markers could not be technically feasible for this study, hlh-14 expression is already assessed for lineaged embryos of all mutant conditions. Therefore, it should be possible for the authors to present the data of hlh-14 expression in Caapp branch for all mutant conditions. Otherwise, the authors should provide a convincing argument of why this analysis is left out of the study.
- The study describes a role of hlh-14 controlling the asymmetric division of the Caap neuroblast, despite its observed expression starting after this cell divides. The authors acknowledge this discrepancy, but fail to provide a hypothesis for a credible molecular mechanism that could explain this observation.
Is the GFP expression of the reporter delayed for some reason? Could hlh-14 expression in Caap be demonstrated by FISH/in situ hybridization probes?

- The authors state that let-19 is an upstream regulator of hlh-14. While this seems true for hlh-14 expression, let-19 mutants do not phenocopy hlh-14 mutants in the regulation of Caapa cell size. The explanation of LET-19 acting on hlh-14 expression maintenance does not correlate well with the assessed time window for let-19 expression requirements (around Caap birth, while hlh-14 expression starts much later). This explanation is not satisfactory for the hierarchical relationship of let-19 and hlh-14.

- In numerous experiments, the expression of both hlh-14 and ceh-63 is assessed with GFP reporters in the same specimens. Although I assume that this is possible thanks to temporal

© 2024. Published by The Company of Biologists under the terms of the Creative Commons Attribution License (https://creativecommons.org/licenses/by/4.0/).

segregation of hlh-14 and ceh-63 reporter expression this is not clearly mentioned in the paper. This is especially confusing because in Fig 1A the expression of hlh-14 is depicted as maintained in the DVC neuron – this would make theoretically impossible to observe hlh-14+/ceh-63- cells. Please clarify this issue.

Specific comments:

- Figure 4: The placement of the experimental conditions is confusing to the reader. In panel A, the hlh-14 mutant should be shown before the fosmid rescue embryo. Similarly, in panel C, hlh-14 mutant should be shown in the middle, as it is correctly shown in the dot plots of panel B.

- Figure 5: In panel A, the stage of the embryos presented in each row should be noted at the left side to improve clarity. In the wild type, two hlh-14 positive cells are shown, while only one appears in Fig 3A, despite both being described as “bean stage”. Please clarify for non-experts for the animal model.

- In panel B, the expression schematic for let-19(t3273) mutant condition is misleading, as it does not show the representative lineage outcome for any of the markers. In fact, it shows the phenotypes observed in the minority of the tracings for each marker. I strongly suggest making two diagrams to show both the most representative situation and the phenotype the authors want to highlight. In the case of let-19(t3200), n=9 for hlh-14 expression and n=7 for dpy-7 and ceh-63 despite the text stating that the three reporters were assessed in the same embryos. Please explain the discrepancy.

- The text for panel E does not state what is the phenotype being assessed for the proportion of embryos (ceh-63 expression?). Moreover, it is not explained why there are a very obvious difference in penetrance between Upshift and Downshift experiments, going from roughly 50% in the former and up to 80% in the latter.

- Figure 6: The mutant conditions should be presented in the same order as in Figure 5 for consistency: first (t3273) and then (t3200). In panel B and C there are n=16 data points for let-19(t3200), including those with and without a burst of hlh-14 expression. If these are the same lineaged embryos from Figure 5B and C, where n=9, authors must explain the sample increase. Similarly, for let-19(t3273) n=5 data points are shown in both neurogenesis phenotype/no phenotype columns: this is not the same proportion shown in Figure 5B and C (4/10 for hlh-14 expression). Please explain discrepancies; It must be clearly stated when the same sample is used, and when different embryos are used.

- Figure S2: In the central rows of panel A, Caapa and Caapp sister cells are shown in separate rows. I think size comparison would be easier if Caapp are presented as an inset (as done in Figure 4).

- Figure S3A: Y axis legend reads “voxesl”, change to “voxels”.

- Figure S8: “The X-axis is the proportion of the sample containing SNP” – this actually describes the Y-axis.

- Line 135: “and find both concomitantly” should read “and find that both concomitantly”

- Line 238: “(normall fated to die)” should read normally

- Line 312-314: “As would be expected, the absolute volume of Caapa is increased in hlh-14 mutants as a result of the change in daughter size ratio, yet in a non-significant manner” should be replaced by “the volume variation of Caapa in hlh-14 mutants is non-significant”.

- Line 434: “We see little effect on the expression of hlh-14/ASCL1 in the DVC neuroblast”: it is not clear to which mutant condition (pig-1 or ham-1) the sentence refers to.

- Line 437: “the production of a DVC neuron is also uninhibited” – phrasing is confusing.

© 2024. Published by The Company of Biologists under the terms of the Creative Commons Attribution License (https://creativecommons.org/licenses/by/4.0/).

- Line 540-542: “Thus, the constraints of developmental timing and polyclonal fate specification mapped over the invariant lineage may combine with the functional requirement for neurons to be small”. This is not proved for DVC neuron, or at least not in this work. I suggest to remove allusions to functional requirement of neuronal size unless is proven.

Reviewer 2

Advance summary and potential significance to field

In this manuscript the authors address the question whether cell size is involved in cell fate regulation - an important question in developmental biology. The study starts by carefully analysing division patterns through 4D-lineage analysis and volumetric quantification in the nervous system and the C lineage in particular. Two divisions that are unequal in size in the branch of the C lineage that precede the formation of the DVC neuroblast were identified and then used as a paradigm to test if cell size alterations brought about by mutating candidate genes affect the fate decisions/behaviour in that lineage.

To this end known regulators of unequal cleavage such as PIG-1 and HAM1 are tested and unequal cleavage in the C lineage are indeed perturbed. The DVC neuroblasts, the last blast cell in that branch of the lineage requires the expression of hlh-14, yet this does not appear to be affected by making these two divisions symmetric. However, the DVC neuroblasts itself re-enters the cell cycle earlier than normal in these conditions and there are alterations in correct cell fate specifications downstream of the Caapa division that normally generates a neuron while the other daughter dies by apoptosis. Instead, in PIG-1 and HAM1 mutants varying outcomes including misspecification, extra divisions etc are observable. Intriguingly, hlh-14 itself appears to regulate unequal division of Caap. To identify upstream regulators of hll-14 this precocious division timing was used to screen a set of mutations which led to the identification of let-19/MDT13 is an upstream regulator of hlh-14 that also affects the unequal divisions observed in the lineage.

All together this study proposes that control of cell size by genes that regulate cell fate through unequal cleavage as a novel mechanism to generate cell size differences in the absence of cell growth.

The ability to address the role of cell size in cell fate determination is currently very limited and the use of C.elegans in which growth is believed to be absent is elegant. The finding that changes in cell size affects cell fate decisions and cell cycle control a couple of divisions downstream is very interesting.

Overall, this is a carefully executed study and the results obtained are in line with the interpretation. This topic is definitively suitable for the readership of Development.

Comments for the author

The results section is very dense in places and the flow here could be increased to make the reading more accessible. The authors may wish to look at the points below.

Absence of growth and cell size modulation:

Are the cells that stem from an abnormally large (or small) precursors also different in size or do they compensate this in some way? This would be interesting to measure in this context. For instance, in pig-1 mutants (eg Fig 2B) Caaa is much smaller than in wild type, yet there are no defects in that lineage. What happens in the case of Caappp? If the initial starting size is affected and there is no growth this should propagate through the lineage.

Is starting from “larger” worse than starting from “smaller”? Are only certain lineages sensitive to cell size change? In the case of extra divisions, are these cells abnormally small?

Let-19 and ham1:

The description of the phenotypes needs tiding up. The paragraphs starting line 345 and 377

© 2024. Published by The Company of Biologists under the terms of the Creative Commons Attribution License (https://creativecommons.org/licenses/by/4.0/).

could be better structured and explained, they are hard to follow. In 40% of let-19 mutants, hlh-14 is no longer expressed and the rest show phenotypes that are rather like pig1 mutants (instead of transformation to hypodermal fate additional divisions etc are observed), but also all are equalising Caa with some having neurogenesis defects, some not and there is transient expression of hlh-14. Can this be untangled more?

Can the different phenotypes observed in let-19 be linked to the presence or absence of hlh-14? Would overexpression of hlh-14 bias the range of phenotypes observed in let-19? Let-19 is expressed in the C lineage and in fact appears to be almost ubiquitously (Fig S9), but apparently not all cells that have let-19 divide unequal in size, so let-19 is not a general regulator of unequal cleavage (as stated in the manuscript) so additional factors in neuroblasts most contribute.

Discussion line 412. Could there be a higher-level conclusion form the present work?

“The work presented in this study provides evidence that cell size alone, as determined by unequal cleavage does not play a major role in the expression of the proneural gene hlh-14/ASCL1 and so the specification of DVC neuroblast fate.” Seems very specific.

The heading line 147, dramatic seems unnecessary.

“Progressive control of unequal cell divisions by neural cell fate regulators determines embryonic neuroblast cell size” I am not sure I understand what “progressive control” refers to. The study does not provide data on how let-19 affects unequal cleavage

Line 319: “his also suggests that HLH-14 must act in Caap, prior to detectable GFP

320 expression in Caapa (Fig. 1A)” not clear without further explanation.

Fig 6A the blue depiction in the schemes at the bottom appears to be mixed up, if I got the data right.

This needs to be cleaned up:

Line 378: “This equalisation is evident in all let-19(t3200) embryo” This equalisation is evident in all let-19(t3200) embryos at the

379 non-permissive temperature of 25°C, including those with transient hlh-14 expression (Fig. 380 6A). In contrast, in let-19(t3273) embryos the cleavage equalisation phenotype correlates with the neurogenesis phenotypes described above (Fig. 5). Embryos lacking any neurogenesis defect in the C lineage also lack a cleavage defect and exhibit wild type unequal cleavages of both Caa and Caap (Fig. 6B,C). For both let-19 alleles the equalisation of the Caa cleavage in affected embryos is significant compared to wild type, and the ratios of 1.17 for t3200 and 1.28 t3273 do not significantly differ from each other

(Fig. 6B).

<u>Reviewer 3</u>

Advance summary and potential significance to field

Cells have characteristic sizes that are associated with their fate and function yet this link between fate and function is only beginning to be explored. In many developmental lineages, asymmetric divisions leading to distinct daughter cell sizes are linked to cell fate decisions, yet why fate and size are linked is unclear. Indeed, precisely why cells have a specific size is often unknown. A number of studies have provided evidence that cell size can influence cell fate but whether these examples are more the exception or the rule is unclear.

In this manuscript, Mullan et al. describe two highly asymmetric divisions involved in a neurogenic lineage in C. elegans embryos and use this lineage to explore the potential links between division size asymmetry and fate specification. They argue that in the case of these divisions, cell size does

© 2024. Published by The Company of Biologists under the terms of the Creative Commons Attribution License (https://creativecommons.org/licenses/by/4.0/).

not directly impact core lineage specification because transcriptional readouts characteristic of correct lineage specification are normal when cell size asymmetry is lost. Instead, they argue that cell size is regulated concomitantly with fate specification pathways, perhaps reflecting the need for neurons to be small, and reveal key roles for several transcriptional regulators in co-regulating fate and size asymmetry.

Specifically, the authors nicely show that hlh-14 expression remains restricted to the correct lineage upon equalization of size asymmetry in Caa or Caap divisions which suggests that size does not directly impact decision making at these two divisions, at least with respect to restricting hlh-14 expression and capacity to direct DVC identity. They go on to define a role in regulating division asymmetry for a number of regulators that impact DVC lineage specification, including a role for HLH-14, suggesting a model in which fate and size are coordinately regulated.
Overall, the manuscript provides a careful and thorough characterisation of the regulation of size and fate within this developmental lineage.

Comments for the author

Key Points:
1. Note this is more a point the authors may wish to expand on the discussion:
While lineage specification defined as a wild-type pattern of hlh-14 expression and the restriction of ceh-63 expression to the DVC neuroblast lineage remains normal in most (but not all) pig-1 embryos, the subsequent division of the neuroblast becomes mis-regulated in the majority of embryos, including loss of apoptosis, multiple/no DVC neurons or reversal in fate between sisters. To what degree do the authors consider that hlh-14 expression is sufficient to 'define proper fate?' At some level, loss of PIG-1 is leading to defects in DVC specification. In other words, the DVC neuroblast has inherited a certain potential reflected by HLH-14, but perhaps because of size changes is rather confused about what it should do. In some sense, if changes in size are affecting downstream pathways, size itself could be considered a 'fate determinant' that acts in parallel to transcription factor identity. Another alternative is that that PIG-1 acts in later divisions as well. Do the authors have any sense if this is the case?
2. 238: HAM-1 is introduced as a positive regulator of PIG-1, yet given the reagents available, I was somewhat surprised they didn't assess whether HAM-1 affects PIG-1 expression in this lineage. It seems unlikely given the respective phenotypes.
3. 474ff: It seems likely that LET-19 is doing something beyond regulating hlh-14 expression as hlh-14 is normal the majority of t2373 embryos (6/10 Fig 5B), yet the majority also undergo symmetric divisions (Fig 6B). Again, this may relate to hlh-14 expression being an incomplete measure of fate/developmental potential.
4. No description of statistical methods, tests used, etc.
5. I struggled to locate certain data cited in the text. In some cases, the data seems missing. In others, I figured it out, but could have been made more clearly.
Some examples where additional quantification or clarity would be useful are listed below:
327: Data for precocious division. How early, how often?
328: Supernumerary divisions in various branches. Can one be more specific or highlight how these were scored? How should we interpret this?
355: all lineage t3200 embryos displace precocious division (Fig 5B). This data is really only presented qualitatively. This statement leads me to assume that in t3273 not all divided precociously?
360: transient burst of expression of variable duration (Fig 5B). Unclear to me how this was quantified. This is only reported as a binary classification. How variable/reliable is this signal? Could this be more frequent than stated due to sensitivity in detection? A detection threshold would explain why HLH-14 is required for asymmetric division before it is detectable in cells.
363: 'never expresses ... ceh-63 when lacking hlh-14' - I assume the 7 in 7/7 reflects the 7/9 lacking hlh-14?

Additional Comments:
* 252: 'Indeed, the absolute size of Caap in those mutants was closer to that of wild types than to other pig-1 mutants in which hlh-14 was expressed (Fig. S5).' -
I found this somewhat curious. How does one square loss of division asymmetry, but WT size of Caap? Is Caa smaller in these embryos and thus could the loss of hlh-14 in these embryos reflect an earlier defect?

© 2024. Published by The Company of Biologists under the terms of the Creative Commons Attribution License (https://creativecommons.org/licenses/by/4.0/).

* 437: 'production of a DVC neuron is also uninhibited' - This is rather odd phrasing. Are the authors implying that DVC specification is normally inhibited and hlh-14 expression relieves this inhibition?
* 917/Figure 5: What do No. 1 and Arrow indicate?
* Figure 5E: Font is nearly unreadable on x-axis
* 'neurogenesis phenotypes described above' - it would be helpful to more clearly define this term as it was unclear to me precisely which phenotypes they are referring to (hlh-1/ceh-63 expression, no or extra neurons/divisions, etc).
* Figure 6: Can the authors comment a bit on the phenotypic differences of let-19(t3200) and let-19(t2373) alleles. I found it surprising that t2373 selectively led to loss of division asymmetry in Caapa/p despite being the less penetrant allele. Could this be simply due to low sample size?
* The genetics and pattern of phenotypes presented are rather complex and layered and there are multiple different phenotypic aspects of lineage specification that do not necessarily correlate. I wonder whether a visual may be useful at the end to summarise findings?

First revision

Author response to reviewers' comments

Response to reviewers

MS ID#: DEVELOP/2022/200981
Old Title: Progressive control of unequal cell divisions by neural cell fate regulators determines embryonic neuroblast cell size
Revised Title: Control of successive unequal cell divisions by neural cell fate regulators determines embryonic neuroblast cell size

We would first like to take the opportunity to thank all three reviewers for their in-depth and insightful comments on our manuscript. Their careful consideration of our manuscript – as evidenced by their detailed reviews – is much appreciated. We welcomed their recognition of the advancements made and the potential significance of our work to the field. We were particularly happy to note their observation that our work is a careful and thorough analysis and that our main conclusions are well supported by the results. All three reviewers also suggested improvements to the text or additional experiments and consequently we have made substantial changes, as outlined in more detail below. Rather than individually list all our changes we have provided two PDF versions of the new manuscript (and supplementary figures and legends), one which includes all tracked text changes. We describe and flag new data and significant figure changes below. We hope these changes have improved the manuscript further, and again express our gratitude to the reviewers for their helpful advice.

Reviewer 1 Advance Summary and Potential Significance to Field

In this paper, the authors describe for the first time that two unequal cell divisions precede the birth of the DVC neuron in the C lineage of C. elegans embryo, a fate that distinguishes this cell from its lineage-related hypodermal cells. The authors set out to answer the question of whether these cell size variations in the DVC progenitors are driving the acquisition of the neural fate in this branch of the lineage. By preventing asymmetric cell division, they convincingly demonstrate that the variation in cell size in the DVC progenitors does not cause cell fate defects, although it can lead to abnormalities in the number and anterior-posterior specification of the neuronal progeny. Finally, the authors discover that two different proneural genes expressed sequentially in the DVC progenitors have an effect on controlling progenitor cell size, besides being required for DVC specification. The authors propose the concomitant regulation of neural fate and cell size as a general mechanism to ensure adequate neuronal size during C. elegans embryonic development, where cell growth mechanisms do not operate. The article is logically structured and easy to follow, and its main conclusions are supported by convincing, well presented data. However, a series of comments should be clarified in order to improve the manuscript.

© 2024. Published by The Company of Biologists under the terms of the Creative Commons Attribution License (https://creativecommons.org/licenses/by/4.0/).

We thank the reviewer for these very positive comments, and we are grateful to the reviewer for noting that our work is well structured with data that convincingly support our conclusions.

Reviewer 1 Comments for the Author

-The authors demonstrate not only that DVC progenitors suffer repeated asymmetric cell divisions to generate a smaller-sized neuron, but also show that this size bias is *evolutionarily conserved across the C lineage of other related nematodes. However, it is not completely clear if the neural fate of the Caapa progeny is also conserved in these related species, or whether only the asymmetries are conserved.*

This is a very interesting point and although it was previously established that certain fates of the progeny of Caapa are indeed conserved in these related species, such as the cell death arising from Caapap (Memar et al. 2019), it was unclear whether Caapaa differentiates into a neuron. We have now re-examined our lineages and performed extended lineaging in all three additional species and compared this to C. elegans. This analysis, which we now present in addition to the previous analysis of cell size in Fig. S1, indicates that based on both position and nuclear morphology Caapaa is likely neuronal in these species. Firstly, at the comma stage of development the Caapaa cell is in an almost identical position in all three species in comparison to C. elegans. This position in C. elegans is known to correspond to the dorsorectal ganglion (which also contains for example the DVA and DVB neurons). Secondly, although the nuclear morphology of DVC in C. elegans is not as granulated as some neuronal nuclei (something we additionally observed for DVA – data not shown), it clearly does not have the classic hypodermal or gut cell nuclear morphology, which look like a 'fried egg' with a prominent nucelolus (see wormatlas cell ID). We find that the nucleus of Caapaa resembles that of DVC in C. elegans. Altogether our additional analysis strongly suggests that the neural fate of Caapaa is likely conserved amongst these nematode species. In further support of this we note that that Caapaa (DVC) and Caappa (PVR) have been reported to be neuronal in another nematode species (Houthoofd et al. 2003). In addition to the updated Fig. S1 we have added a sentence reporting this new data in the first results section ("Two unequal cleavages…."). We think that further reporter-gene based analysis in these species is beyond the scope of this manuscript.

- The authors discuss that the proneural genes hlh-14 and let-19 act concomitantly to control the formation of a DVC neuron of the adequate size and argue for the requirement of cellular mechanisms that control cell size at the level of cell division in an embryo where cell growth/shrinkage is not permitted. However, although the effect of abnormal cell size in cell cycle and neuronal progeny number is shown, the possible effects of a larger neuroblast in function/morphology of the resulting DVC neuron are not experimentally tackled or discussed.

- DVC neuron size (volume) is not measured. If technically possible, this measurement should be quantified for hlh-14 and let-19 mutants.
- An assessment of adult morphology and function of DVC in mutant animals, if technically possible, would provide a better picture of the relevance of the neuroblast size control processes described in this article.

It is technically not possible to measure the size of Caapaa (DVC) by DIC as the cells are too small in comparison to the resolution of the DIC images. We have tried but the variability in our measurements, caused by the difficulty of determining the exact position of the membrane, did not permit us to generate informative data. We have however been able to measure the nuclear size of Caapaa (as a proxy for cell size) using a translational hlh-14::gfp reporter. Although the nucleus is of course smaller than the cell, it is much easier to disambiguate the outline of nucleus in these fluorescent images in comparison to the cell membrane in DIC images. However, since the hlh-14 reporters we have are both rescuing translational fusions we have only been able to do this in pig-1 mutants. This new data is presented in Fig. 4 and Fig. S7, new figures generated for this revision. These experiments now allow us to demonstrate that the

© 2024. Published by The Company of Biologists under the terms of the Creative Commons Attribution License (https://creativecommons.org/licenses/by/4.0/).

terminal division of Caapa, to generate Caapaa (DVC) and Caapap (cell death) is unequal and that thiscleavage asymmetry is also dependent on pig-1. We have also clarified this phenotype in the text with in a now expanded results section ("pig-1 and ham-1 affect the asymmetric division of the DVC neuroblast"). It is highly likely that symmetrisation of this terminal division is the cause of defects in cell death/ectopic DVC neurons we previously observed. Our interpretation is that at this terminal division size does indeed affect the ability of a cell to die (being too large inhibits cell death) and this is very consistent with the previously reported roles of pig-1 at terminal divisions that generate a neuron and a cell death (see manuscript text for references).

This effect on the terminal division in pig-1 mutants acts to reduce the final size of Caapaa (DVC) such that despite the increase in overall size generated by the defects in the unequal cleavafes of Caa and Caap, the actual final size of DVC (as measured using nuclear size as a proxy for cell size) is not significantly different to wildtype (Fig. S7). This precludes any functional studies. In any case, the number of well-described pleiotropic defects in pig-1 mutant animals, such as the generation of ectopic neurons due to inappropriate survival of apoptotic sister cells (see references in manuscript) and the lack of a well-described functional role for DVC also makes functional experiments and their interpretation almost impossible. The tools to generate DVC-specific knockout of pig-1 do not currently exist. We have however now examined more carefully the morphology of DVC in pig-1 mutant larvae and do not observe any obvious defects. This data, which is now presented in a new figure, Fig. S4, further strengthens our conclusion that the earlier defects in the unequal divisions do not affect the specification of the DVC neuron. We are unable to examine larval/adult morphology of Caapaa (DVC) in hlh-14 and let-19 mutants as they are early larval lethal and embryonic lethal respectively.

-The Caap neuroblast produces two neurons, DVC from the Caapa neuroblast and PVR from the Caapp neuroblast, and in both cases the proneural gene hlh-14 is activated.
However, how the different mutant conditions (pig-1, ham-1, hlh-14 and let-19) affect the fate of PVR neuron is not analyzed or discussed. The analysis of PVR fate would help generalize the conclusions of the study regarding the effect of cell size on neuronal fate and the let-19 requirement for downstream hlh-14 expression. Understanding that a study of PVR fate with specific markers could not be technically feasible for this study, hlh-14 expression is already assessed for lineaged embryos of all mutant conditions. Therefore, it should be possible for the authors to present the data of hlh-14 expression in Caapp branch for all mutant conditions. Otherwise, the authors should provide a convincing argument of why this analysis is left out of the study.

We agree that an analysis of PVR cell fate would help to generalise our conclusions that the unequal divisions of Caa and Caap have no effect on the later specification of both DVC and PVR cell fate. We did not initially assess the expression of hlh-14 in PVR because the hlh-14::gfp [gmIs20] transgene lacks PVR expression. This is in contrast to a fosmid-based hlh-14^{fos}::gfp reporter which is expressed in PVR. This expression difference is now illustrated in Fig. S8, a new figure. We have now examined the expression of this fosmid-based hlh-14 reporter in two different pig-1 mutants and find that its expression is entirely normal in PVR in these mutant backgrounds. This new data is now presented in a modified version of Fig. 3 and described in the results text in the section 'Equalisation of the Caa or Caap blastomere has little effect on hlh-14 expression….'. Fig. 3 now only contains our analysis of hlh-14 expression. Our new analysis of the terminal division of the DVC neuroblast and our previous analysis of DVC specification has been moved to Fig. 4, a new figure. We have not assessed the regulation of hlh-14 in let-19 mutants as we feel that our analysis already strongly demonstrates that let-19 is an upstream regulator of hlh- 14 in the DVC branch. Our new analysis does however further validate and generalise our conclusion that size alone does not affect proneural gene expression in this lineage.

-The study describes a role of hlh-14 controlling the asymmetric division of the Caap neuroblast, despite its observed expression starting after this cell divides. The authors acknowledge this discrepancy, but fail to provide a hypothesis for a credible molecular mechanism that could explain this observation. Is the GFP expression of the reporter delayed for some reason? Could hlh-14 expression in Caap be demonstrated by FISH/in situ hybridization probes?

© 2024. Published by The Company of Biologists under the terms of the Creative Commons Attribution License (https://creativecommons.org/licenses/by/4.0/).

We agree that an analysis of hlh-14 expression via smFISH would be a very elegant way to demonstrate expression in Caap, prior to the observed expression of the hlh-14::gfp [gmIs20] transgene. However, due to very frustrating probe supply issues we have been unable to perform these experiments. Instead, we first analysed the expression of hlh-14 as described in an embryonic scRNAseq dataset (Packer et al 2019) and found that transcripts are detected immediately upon the birth of Caapa (data not shown). The absence of transcript detection in Caap may well be due to the fact that there are difficulties in disambiguating Ca vs Cp lineages (in fact until proneural/neural gene expression is readily detectable) due to the bilateral symmetry of these two lineages. We therefore sought another way to monitor hlh-14 expression and decided to examine a strain containing a fosmid-based hlh-14 reporter. This strain recapitulates more aspects of hlh-14 expression than the gmIs20 transgene (Fig. S8). We find that in contrast to the gmIs20 transgene, the fosmid-based reporter displays two waves of expression in Caapa, the first of which begins as early as 11 minutes after birth of Caapa. Given the ~30 min maturation time of GFP this expression can only reasonably be explained by hlh-14 transcription in Caap. We now describe this at the end of the 'The Caap blastomere cleavage is affected…' results section.

-The authors state that let-19 is an upstream regulator of hlh-14. While this seems true for hlh-14 expression, let 19 mutants do not phenocopy hlh-14 mutants in the regulation of Caapa cell size. The explanation of LET-19 acting on hlh-14 expression maintenance does not correlate well with the assessed time window for let-19 expression requirements (around Caap birth, while hlh-14 expression starts much later). This explanation is not satisfactory for the hierarchical relationship of let-19 and hlh-14

There is clearly a hierarchical relationship as hlh-14 expression is lost in let-19 mutants. Fig. 7 also shows that the unequal division of Caap in let-19(t2373) mutants is affected in those embryos that completely lack hlh-14 expression. Additionally, our new analysis of an hlh-14 fosmid-based reporter reveals there are in fact two phases of hlh-14 expression observable in Caapa (Fig. S8). The is observable in Caapa as few as 11 mins following the division of Caap. Given the maturation time of GFP this strongly suggests expression is initiated in Caap, where it must act to regulate the unequal division. The second phase is initiated ~40mins into the Caapa cell cycle the Caapa cell cycle. Again, given the maturation time of GFP it is likely that the second wave of hlh-14 transcription begins earlier in the Caapa cell-cycle at a time consistent with our temperature-shift experiments. Our interpretation, which has now been modified in the discussion text, is that let-19 is not required for the first phase of expression but is required for the second phase of expression. We also note that we make no claim that the regulation of either wave of hlh- 14 expression by let-19 is direct.

-In numerous experiments, the expression of both hlh-14 and ceh-63 is assessed with GFP reporters in the same specimens. Although I assume that this is possible thanks to temporal segregation of hlh-14 and ceh-63 reporter expression, this is not clearly *mentioned in the paper. This is especially confusing because in Fig 1A the expression of hlh-14 is depicted as maintained in the DVC neuron – this would make theoretically impossible to observe hlh-14+/ceh-63- cells. Please clarify this issue.*

We thank the reviewer for pointing out our lack of clarity here. The expression of hlh-14 and ceh-63 is temporally non-overlapping in DVC and we have altered Fig. 1A accordingly. Expression of the two transgenes can also be disambiguated because expression of the hlh-14 transgene is nuclear and expression of the ceh-63 transgene is cytoplasmic. We have clarified this in the text at the end of the 'pig-1 and ham-1 affect asymmetric…' results section.

Specific comments:

-Figure 4: The placement of the experimental conditions is confusing to the reader. In panel A, the hlh-14 mutant should be shown before the fosmid rescue embryo. Similarly, in panel C, hlh-14 mutant should be shown in the middle, as it is correctly shown in the dot plots of panel B.

We have corrected this and note that this data, previously in Fig. 4 is now in Fig. 5.

© 2024. Published by The Company of Biologists under the terms of the Creative Commons Attribution License (https://creativecommons.org/licenses/by/4.0/).

-Figure 5: In panel A, the stage of the embryos presented in each row should be noted at the left side to improve clarity. In the wild type, two hlh-14 positive cells are shown, while only one appears in Fig 3A, despite both being described as “bean stage”. Please clarify for non-experts for the animal model.

We have now clarified the staging throughout the manuscript and in all figures (where necessary) by referring to the total number of cells that have been generated from the C blastomere. So C2 = Ca/Cp stage etc. We have also illustrated this staging scheme in Fig. 1A in relation to actual developmental time (for the reader to refer back to if they are unsure about staging). This also avoids the need to use time or C. elegans specific staging nomenclature such as ‘bean’ and is in fact also more precise/descriptive of the exact stage in question.

-In panel B, the expression schematic for let-19(t3273) mutant condition is misleading, as it does not show the representative lineage outcome for any of the markers. In fact, it shows the phenotypes observed in the minority of the tracings for each marker. I strongly suggest making two diagrams to show both the most representative situation and the phenotype the authors want to highlight. In the case of let-19(t3200), n=9 for hlh-14 expression and n=7 for dpy-7 and ceh-63, despite the text stating that the three reporters were assessed in the same embryos. Please explain the discrepancy.

We thank the reviewer for pointing these discrepancies out and agree that panel B was misleading. As a result of this comment we have gone back and re-analysed all our data concerning the phenotypes of let-19 mutants. The data presented are now entirely from 4D-lineaged embryos, as opposed to a combination of lineaged and staged embryos. This has led to a complete re-working of what was Fig. 5 and Fig. 6 to generate two new figures, Fig. 6 and Fig. 7. As now stated in the figure legend for Fig. 6, single transgenes were assessed in both t3273 and t3200 mutants, with all three assessed concomitantly in a subset of t3200 mutants. For clarity the concomitant assessment of the three markers in t3200 is now only highlighted to demonstrate temporal and causal links between the expression of the markers, rather than being presented as a separate dataset to scoring conducted with single transgene carrying strains. These previous discrepancies are now either absent or explicitly explained in the main text or figure legends. Clarity for the reader is therefore much improved.

-The text for panel E does not state what is the phenotype being assessed for the proportion of embryos (ceh-63 expression?). Moreover, it is not explained why there are a very obvious difference in penetrance between Upshift and Downshift experiments, going from roughly 50% in the former and up to 80% in the latter.

The reviewer is correct and it is indeed ceh-63 expression that was assessed. We have modified the x-axis of the graph (now Fig. 6E) to reflect this. We suspect the difference in penetrance between upshifts and downshifts relates to the stability of LET-19 protein. In the upshifts there is the possibility that wildtype protein is still around in the cells, which is not the case with the downshifts.

-Figure 6: The mutant conditions should be presented in the same order as in Figure 5 for consistency: first (t3273) and then (t3200). In panel B and C there are n=16 data points for let-19(t3200), including those with and without a burst of hlh-14 expression. If these are the same lineaged embryos from Figure 5B and C, where n=9, authors must explain the sample increase. Similarly, for let- 19(t3273) n=5 data points are shown in both neurogenesis phenotype/no phenotype columns: this is not the same proportion shown in Figure 5B and C (4/10 for hlh-14 expression). Please explain discrepancies; It must be clearly stated when the same sample is used, and when different embryos are used.

We again agree with the reviewer here, there was a lack of clarity throughout the manuscript as to which samples were used when and indeed also the nature of some of the wild type genotypes (and whether they do or don’t contain the transgenes we also use to assess gene expression). We have now made substantial changes to clarify this throughout the manuscript, within the text and also the figure legends. In addition, we have added a new methods section ‘Statistical analysis

© 2024. Published by The Company of Biologists under the terms of the Creative Commons Attribution License (https://creativecommons.org/licenses/by/4.0/).

and genotypes' which clarifies the genotypes used throughout the manuscript and refer readers to it in every appropriate figure legend. We have also generated a new figure, Fig. S11 which demonstrates there are no statistical differences in cell size ratios across the different wildtype genotypes we used (with different transgenes in the background). We thank the reviewer for this comment and think the manuscript is now much clearer in this regard.

-Figure S2: In the central rows of panel A, Caapa and Caapp sister cells are shown in separate rows. I think size comparison would be easier if Caapp are presented as an inset (as done in Figure 4).

We actually think it is easier as separate rows and have instead modified what is now Fig. 5 to correspond to what is now Fig. S1

-Figure S3A: Y axis legend reads "voxesl", change to "voxels".

Done

-Figure S8: "The X-axis is the proportion of the sample containing SNP" – this actually describes the Y-axis.

Done

-Line 135: "and find both concomitantly" should read "and find that both concomitantly"

Done

-Line 238: "(normall fated to die)" should read normally

Done

-Line 312-314: "As would be expected, the absolute volume of Caapa is increased in hlh- 14 mutants as a result of the change in daughter size ratio, yet in a non-significant manner" should be replaced by "the volume variation of Caapa in hlh-14 mutants is non- significant".

Done

-Line 434: "We see little effect on the expression of hlh-14/ASCL1 in the DVC neuroblast": it is not clear to which mutant condition (pig-1 or ham-1) the sentence refers to.

Done

-Line 437: "the production of a DVC neuron is also uninhibited" – phrasing is confusing.

Done

-Line 540-542: "Thus, the constraints of developmental timing and polyclonal fate specification mapped over the invariant lineage may combine with the functional requirement for neurons to be small". This is not proved for DVC neuron, or at least not in this work. I suggest to remove allusions to functional requirement of neuronal size unless is proven.

Done

******* Reviewer 2 Advance Summary and Potential Significance to Field:**

In this manuscript the authors address the question whether cell size is involved in cell fate regulation – an important question in developmental biology. The study starts by carefully analysing division patterns through 4D-lineage analysis and volumetric quantification in the nervous system and the C lineage in particular. Two divisions that are unequal in size in the branch

© 2024. Published by The Company of Biologists under the terms of the Creative Commons Attribution License (https://creativecommons.org/licenses/by/4.0/).

of the C lineage that precede the formation of the DVC neuroblast were identified and then used as a paradigm to test if cell size alterations brought about by mutating candidate genes affect the fate decisions/behaviour in that lineage.

To this end known regulators of unequal cleavage such as PIG-1 and HAM1 are tested and unequal cleavage in the C lineage are indeed perturbed. The DVC neuroblasts, the last blast cell in that branch of the lineage, requires the expression of hlh-14, yet this does not appear to be affected by making these two divisions symmetric. However, the DVC neuroblasts itself re-enters the cell cycle earlier than normal in these conditions and there are alterations in correct cell fate specifications downstream of the Caapa division that normally generates a neuron while the other daughter dies by apoptosis. Instead, in PIG-1 and HAM1 mutants varying outcomes including misspecification, extra divisions etc are observable. Intriguingly, hlh-14 itself, appears to regulate unequal division of Caap. To identify upstream regulators of hll-14 this precocious division timing was used to screen a set of mutations which led to the identification of let-19/MDT13 is an upstream regulator of hlh-14 that also affects the unequal divisions observed in the lineage.

All together this study proposes that control of cell size by genes that regulate cell fate through unequal cleavage as a novel mechanism to generate cell size differences in the absence of cell growth.

The ability to address the role of cell size in cell fate determination is currently very limited and the use of C.elegans in which growth is believed to be absent is elegant. The finding that changes in cell size affects cell fate decisions and cell cycle control a couple of divisions downstream is very interesting.

Overall, this is a carefully executed study and the results obtained are in line with the interpretation. This topic is definitively suitable for the readership of Development.

We thank the reviewer for these very positive comments, and we are grateful to the reviewer for noting that the our interpretation is consistent with our results. We also thank the reviewer for noting that the topic of our study is suitable for the readership of Development.

Reviewer 2 Comments for the Author:

The results section is very dense in places and the flow here could be increased to make the reading more accessible. The authors may wish to look at the points below.

We have made a large number of changes throughout the manuscript, but also in the figures and figure legends that we think addresses the reviewers concerns (please see the PDF with tracked changes). We note that Reviewer 1 actually stated they found the manuscript 'easy to follow' but we nevertheless identified places where we also felt the results were too dense. We thank the reviewer for this comment and believe we have now made the reading more accessible throughout.

Absence of growth and cell size modulation:

- Are the cells that stem from an abnormally large (or small) precursors also different in size or do they compensate this in some way? This would be interesting to measure in this context. For instance, in pig-1 mutants (eg Fig 2B) Caaa is much smaller than in wild type, yet there are no defects in that lineage. What happens in the case of Caappp? If the initial starting size is affected and there is no growth this should propagate through the lineage.

The reviewer is correct in that if initial starting size is affected this size difference is indeed propagated through the lineage. Some of the data in support of this was originally presented in Fig. S3 and described in the original manuscript. These data are now presented in an improved version of this figure, now Fig. S2, in which the absolute sizes of the Caap and Caapa are displayed across all the genotypes tested. One can easily see for example that when Caap is larger is in let-19 mutants, this leads to an increase in size of Caapa (even though the division of Caap is not affected in let-19 mutants). We do not observe any compensation in this regard and

© 2024. Published by The Company of Biologists under the terms of the Creative Commons Attribution License (https://creativecommons.org/licenses/by/4.0/).

so our results are entirely consistent with an absence of cell growth. This propagation through the lineage is described in several places in the manuscript. We have now also performed additional cell size analysis during the terminal divison of the DVC neuroblast in pig-1 mutants, using nuclear size as a proxy for cell size (Fig. 4 and Fig. S7). This analysis demonstrates that DVC itself is not significantly larger in pig-1 mutant animals. However, the reason for this is that the cleavage of the terminal divison of the DVC neuroblast is also symmetrised in pig-1 mutant animals in a manner that actually reduces cell size again. Our interpretation of this data is that pig-1 is also required for the unequal cleavage of the DVC neuroblast (see in addition our response to Reveiwer 1 above). We do not think this a compensatory mechanism and it does not involve some sort of cell shrinkage mechanism as the symmetrisation of the division is observable immediately after the cleavage has occurred. We think that the analysis of other lineages, such as Caa, is beyond the scope of this work.

- Is starting from “larger” worse than starting from “smaller”? Are only certain lineages sensitive to cell size change? In the case of extra divisions, are these cells abnormally small?

Although we are not quite sure what the reviewer means here by ‘worse’ these are all very intriguing questions, but we think they are beyond the scope of this current work. We have however noticed, and this is now mentioned in the discussion, that there is a trend in pig-1 mutants for the extra divisions we observe of Caapa (the DVC neuroblast) to be correlated with an increase in size of the DVC neuroblast – the largest ones are those that divide again. We are intrigued that this could reflect a compensatory mechanism that relies on cell division control rather than cell growth/shrinkage and is something we wish to investigate more in the future.

Let-19 and ham1:

- The description of the phenotypes needs tiding up. The paragraphs starting line 345 and 377 could be better structured and explained, they are hard to follow. In 40% of let-19 mutants, hlh-14 is no longer expressed and the rest show phenotypes that are rather like pig1 mutants (instead of transformation to hypodermal fate additional divisions etc are observed), but also all are equalising Caa with some having neurogenesis defects, some not and there is transient expression of hlh-14. Can this be untangled more?

We agree with the reviewer that the description of let-19 mutant phenotypes was unclear and quite confusing in places. Particularly since we observe slightly different phenotypes in the two different let-19 alleles. For example, the extra divisions we observe in let-19 mutants are restricted to t3273 and are not restricted to the DVC lineage. Reviewer 1 also commented on the lack of clarity in our descriptions of let-19 mutant phenotypes (see our response above). To address these concerns, we have substantially altered the results sections referring to let-19 and have generated two new figures (Fig. 6 and Fig. 7) that we think present our let-19 data much more clearly.

Can the different phenotypes observed in let-19 be linked to the presence or absence of hlh-14? Would overexpression of hlh-14 bias the range of phenotypes observed in let-19? Let-19 is expressed in the C lineage and in fact appears to be almost ubiquitously (Fig S9), but apparently not all cells that have let-19 divide unequal in size, so let-19 is not a general regulator of unequal cleavage (as stated in the manuscript) so additional factors in neuroblasts most contribute.

Yes, the different phenotypes observed in let-19, particularly in the t3273 allele, can indeed be correlated to the presence/absence of hlh-14. We have clarified this in our re- worked Fig. 6 and Fig. 7 and in the let-19 results section. As part of the mediator complex, LET-19 is a ‘general’ factor required for transcriptional activation/repression. We did not mean to imply let-19 itself is solely a regulator of unequal cleavage and the cell-specificity in relation to unequal cleavage must indeed come from TF-binding partners.

Discussion line 412. Could there be a higher-level conclusion form the present work?

“The work presented in this study provides evidence that cell size alone, as determined by unequal cleavage, does not play a major role in the expression of the proneural gene hlh-

© 2024. Published by The Company of Biologists under the terms of the Creative Commons Attribution License (https://creativecommons.org/licenses/by/4.0/).

14/ASCL1 and so the specification of DVC neuroblast fate." Seems very specific.

We have now added "We can therefore conclude that in a variety of different contexts size does not affect cell fate specification".

The heading line 147, dramatic seems unnecessary.

Removed.

"Progressive control of unequal cell divisions by neural cell fate regulators determines embryonic neuroblast cell size" I am not sure I understand what "progressive control" refers to. The study does not provide data on how let 19 affects unequal cleavage

By progressive we were trying to allude to the fact that the division of Caa is regulated by let-19 and the division of Caapa by hlh-14. Both of these genes are additionally required for the correct cell fate specification in this lineage and the ultimate production of the DVC neuron. We were not trying to suggest we have demonstrated the mechanism by which let-19 affects unequal cleavage (although this an extremely interesting question - same for hlh-14). However, we agree with the reviewer that 'progressive' is ambiguous and have altered the title to 'Control of successive divisions….'.

Line 319: "his also suggests that HLH-14 must act in Caap, prior to detectable GFP expression in Caapa (Fig. 1A)" not clear without further explanation.

Reviewer 1 also had concerns about this statement - please see our response to reviewer 1 above as we now additionally examined a fosmid-based hlh-14 reporter that allows us to shed more light on this issue (Fig S8). We have modified both this statement in the results section but also the discussion text accordingly.

Fig 6A the blue depiction in the schemes at the bottom appears to be mixed up, if I got the data right.

Corrected.

This needs to be cleaned up:

Line 378: "This equalisation is evident in all let-19(t3200) embryo" This equalisation is evident in all let-19(t3200) embryos at thenon-permissive temperature of 25°C, including those with transient hlh-14 expression (Fig. 380 6A). In contrast, in let-19(t3273) embryos the cleavage equalisation phenotype correlates with the neurogenesis phenotypes described above (Fig. 5). Embryos lacking any neurogenesis defect in the C lineage also lack a cleavage defect and exhibit wild type unequal cleavages of both Caa and Caap (Fig. 6B,C). For both let-19 alleles the equalisation of the Caa cleavage in affected embryos is significant compared to wild type, and the ratios of 1.17 for t3200 and 1.28 t3273 do not significantly differ from each other (Fig. 6B).

As described above we have undertaken substantial changes in both the text and the figures that clarify our let-19 results.

******* Reviewer 3 Advance Summary and Potential Significance to Field:**

Cells *have characteristic sizes that are associated with their fate and function, yet this link between fate and function is only beginning to be explored. In many developmental lineages, asymmetric divisions leading to distinct daughter cell sizes are linked to cell fate decisions, yet why fate and size are linked is unclear. Indeed, precisely why cells have a specific size is often unknown. A number of studies have provided evidence that cell size can influence cell fate, but whether these examples are more the exception or the rule is unclear.*

© 2024. Published by The Company of Biologists under the terms of the Creative Commons Attribution License (https://creativecommons.org/licenses/by/4.0/).

In this manuscript, Mullan et al. describe two highly asymmetric divisions involved in a neurogenic lineage in C. elegans embryos and use this lineage to explore the potential links between division size asymmetry and fate specification. They argue that in the case of these divisions, cell size does not directly impact core lineage specification because transcriptional readouts characteristic of correct lineage specification are normal when cell size asymmetry is lost. Instead, they argue that cell size is regulated concomitantly with fate specification pathways, perhaps reflecting the need for neurons to be small, and reveal key roles for several transcriptional regulators in co-regulating fate and size asymmetry.

Specifically, the authors nicely show that hlh-14 expression remains restricted to the correct lineage upon equalization of size asymmetry in Caa or Caap divisions, which suggests that size does not directly impact decision making at these two divisions, at least with respect to restricting hlh-14 expression and capacity to direct DVC identity. They go on to define a role in regulating division asymmetry for a number of regulators that impact DVC lineage specification, including a role for HLH-14, suggesting a model in which fate and size are coordinately regulated. Overall, the manuscript provides a careful and thorough characterisation of the regulation of size and fate within this developmental lineage.

We thank the reviewer for their very positive comments and were very pleased that they found this work both careful and thorough.

Reviewer 3 Comments for the Author:

Key Points:

1. Note this is more a point the authors may wish to expand on the discussion:
While lineage specification defined as a wild-type pattern of hlh-14 expression and the restriction of ceh-63 expression to the DVC neuroblast lineage remains normal in most (but not all) pig-1 embryos, the subsequent division of the neuroblast becomes mis- regulated in the majority of embryos, including loss of apoptosis, multiple/no DVC neurons or reversal in fate between sisters. To what degree do the authors consider that hlh-14 expression is sufficient to 'define proper fate?' At some level, loss of PIG-1 is leading to defects in DVC specification. In other words, the DVC neuroblast has inherited a certain potential reflected by HLH-14, but perhaps because of size changes is rather confused about what it should do. In some sense, if changes in size are affecting downstream pathways, size itself could be considered a 'fate determinant' that acts in parallel to transcription factor identity. Another alternative is that that PIG-1 acts in later divisions as well. Do the authors have any sense if this is the case?

The reviewers last speculation, that pig-1 acts during the division of the DVC neuroblast was in fact spot on! We now provide data in an entirely new figure (Fig. 4) that indicates this division is normally unequal, that it is symmetrised in pig-1 mutants and this leads to loss of apoptosis and asymmetric cell fate defects (see also our response to Reviewer 1 above). This may also be the reason that ectopic divisions are observed in this branch in the pig-1 mutants. An alternative possibility, which we now speculate on briefly in the discussion, is that the increased size is somehow sensed by the cell and the ectopic divisions are a compensatory mechanism. Consistent with this there is a clear trend for the larger DVCs to be those that undergo an ectopic round of division. We now present this data in Fig. S11.

2. 238: HAM-1 is introduced as a positive regulator of PIG-1, yet given the reagents available, I was somewhat surprised they didn't assess whether HAM-1 affects PIG-1 expression in this lineage. It seems unlikely given the respective phenotypes.

It is exactly for this reason that we did not assess whther ham-1 regulates pig-1 expression in this lineage. We also feel that an assessment of this sort is beyond the scope of this piece of work.

3. 474: It seems likely that LET-19 is doing something beyond regulating hlh-14expression as hlh-14 is normal the majority of t2373 embryos (6/10 Fig 5B), yet the majority also undergo symmetric divisions (Fig 6B). Again, this may relate to hlh-14 expression being an incomplete measure of fate/developmental potential.

© 2024. Published by The Company of Biologists under the terms of the Creative Commons Attribution License (https://creativecommons.org/licenses/by/4.0/).

Actually, this is not the case - we find that the expression/lack of expression of hlh-14 and unequal/equal divisions are correlated in t3273 embryos (Fig. 7B). This was not very clear in our original manuscript but our re-analysis of let-19 mtuants and our new figures should now clarify this for the reader. However, even those with hlh-14 do sometimes undergo ectopic divisions (Fig. 7D). As let-19 is a component of a very broadly used transcriptional regulatory complex some pleiotropy is likely. Moreover, this effect is restricted to the t3273 allele and is not observed in t3200 - indicating allele-specific pleiotropies.

4. No description of statistical methods, tests used, etc.

We apologise for this poor oversight and have now added a specific methods section.

5. I struggled to locate certain data cited in the text. In some cases, the data seems missing. In others, I figured it out, but could have been made more clearly.
Some examples where additional quantification or clarity would be useful are listed below:

- 327: Data for precocious division. How early, how often?

We have now analysed division timing of the DVC blastomere in much more detail and present this data in Fig. 2, 5 and 7 (on the lineage diagrams). The 'raw' data is provided in Fig. S3.

- 328: Supernumerary divisions in various branches. Can one be more specific or highlight how these were scored? How should we interpret this?

We have now indicated this data much more clearly, both on Fig. 7D. Ectopic divisions were observed and scored via manual 4D-lineaging (as was most data in the paper).

- 355: all lineage t3200 embryos displace precocious division (Fig 5B). This data is really only presented qualitatively. This statement leads me to assume that in t3273 not all divided precociously?

Please see our response to your previous-but-one comment above.

- 360: transient burst of expression of variable duration (Fig 5B). Unclear to me how this was quantified. This is only reported as a binary classification. How variable/reliable is this signal? Could this be more frequent than stated due to sensitivity in detection? A detection threshold would explain why HLH-14 is required for asymmetric division before it is detectable in cells.

We have clarified this further in the text, it was done via 4D-lineaigng and indeed is a binary classification. It is very possible that threshold issues could preclude us observing it more frequently. Our new analysis of the fosmid-based hlh-14 reporter is in fact consistent with hlh-14 transcription in Caap.

363: 'never expresses ... ceh-63 when lacking hlh-14' - I assume the 7 in 7/7 reflects the 7/9 lacking hlh-14?

We have clarified this in the text.

Additional Comments:

* 252: 'Indeed, the absolute size of Caap in those mutants was closer to that of wild types than to other pig-1 mutants in which hlh-14 was expressed (Fig. S5).' - I found this somewhat curious. How does one square loss of division asymmetry, but WT size of Caap? Is Caa smaller in these embryos and thus could the loss of hlh-14 in these embryos reflect an earlier defect?

© 2024. Published by The Company of Biologists under the terms of the Creative Commons Attribution License (https://creativecommons.org/licenses/by/4.0/).

We have now analysed the terminal division of the DVC neuroblast in much more detail (Fig. 4 and Fig. S7) clarifying this discrepancy.

* 437: 'production of a DVC neuron is also uninhibited' - This is rather odd phrasing. Are the authors implying that DVC specification is normally inhibited and hlh-14 expression relieves this inhibition?

This has now been rephrased to improve clarity (see the PDF containing tracked changes)

* 917/Figure 5: What do No. 1 and Arrow indicate?

This is now indicated in the figure legends.

* Figure 5E: Font is nearly unreadable on x-axis

We have increased font sizes on our new figures.

* 'neurogenesis phenotypes described above' - it would be helpful tomore clearly define this term as it was unclear to me precisely which phenotypes they are referring to (hlh- 1/ceh-63 expression, no or extra neurons/divisions, etc).

This relates back to an earlier comment – we agree that 'neurogenesis phenotype' was vague and now refer specifically to embryos expressing/not expressing hlh-14.

* Figure 6: Can the authors comment a bit on the phenotypic differences of let-19(t3200) and let-19(t2373) alleles. I found it surprising that t2373 selectively led to loss of division asymmetry in Caapa/p despite being the less penetrant allele. Could this be simply due to low sample size?

We do not agree that the let-19(t3273) allele leads to a selective loss of Caapa/p division asymmetry and we hope this is clearer in our new let-19 figures (Fig. 6 and Fig 7). In fact what we observe is that when hlh-14 expression is lost there is a very strong effect on the division asymmetry of Caaa/p and a more variable effect on the division of Caapa/p. When hlh-14 is expressed we see no significant defects in unequal divisions. As partly discussed above, we suspect that the phenotypic differences of the two alleles may well be due to different effects in TF-binding partners. LET-19 is part of a very general transcriptional regulatory complex - the Mediator and so allele-specific phenotypes are very plausible.

* The genetics and pattern of phenotypes presented are rather complex and layered and there are multiple different phenotypic aspects of lineage specification that do not necessarily correlate. I wonder whether a visual may be useful at the end to summarise findings?

We think our new experimental analysis and the improvements we have made to the clarity of both the text and the figures obviates the need for this kind summary.

Second decision letter

MS ID#: DEVELOP/2022/200981

MS TITLE: Control of successive unequal cell divisions by neural cell fate regulators determines embryonic neuroblast cell size

AUTHORS: Thomas W Mullan, Terry Felton, Janis Tam, Osama Kasem, Justina Yeung, Nadin Memar, Ralf Schnabel, and Richard J Poole

ARTICLE TYPE: Research Article

© 2024. Published by The Company of Biologists under the terms of the Creative Commons Attribution License (https://creativecommons.org/licenses/by/4.0/).

I am happy to tell you that your manuscript has been accepted for publication in Development, pending our standard ethics checks.

Reviewer 1

Advance summary and potential significance to field

The three reviewers were all quite positive about the advances in the paper. The way the authors responded to the multiple suggestions from the reviewers made the paper easier to read and more rigorous.
The paper can now be published

Comments for the author

The paper is now fine and should be published

© 2024. Published by The Company of Biologists under the terms of the Creative Commons Attribution License (https://creativecommons.org/licenses/by/4.0/).
